# Mechanical Performance of Reinforced Epoxy-Grouted Concrete Interlayer Systems Under Complex Loading and Wet–Dry Cycles

**DOI:** 10.3390/ma19163467

**Published:** 2026-08-17

**Authors:** Yidang Pan, Jingyuan Ma

**Affiliations:** 1 School of Civil Engineering and Architecture, Zhejiang University of Science & Technology, Hangzhou 310023, China; 15510533606@163.com; 2 Institute of Hypergravity Science and Technology, Zhejiang University, Hangzhou 310058, China

**Keywords:** epoxy-grouted concrete, reinforced epoxy, mechanical performance, digital image correlation (DIC)

## Abstract

**Highlights:**

- Reinforcing materials incorporation transforms brittle epoxy failure into progressive quasi-brittle damage.
- Optimal reinforcement type for concrete–epoxy interlayer systems depends on the dominant loading mode.
- DIC analysis confirmed the direct correlation between interface adhesion and macroscopic failure pathways.

**Abstract:**

Concrete structures are prone to cracking during service, and epoxy grouting is a widely adopted technique for structural intervention. However, the inherent brittleness and poor durability of neat epoxy under complex loading and environmental exposure remain critical challenges. This study systematically evaluates the mechanical performance of epoxy-grouted concrete interlayer systems modified by carbon fiber (CF), glass fiber (GF), and polyethylene microspheres (PE). A comprehensive experimental program was conducted, including compression, three-point bending, and Brazilian splitting tests at three loading angles, combined with digital image correlation for surface strain monitoring. The effects of grout thickness and wet–dry cycles were systematically investigated. Results demonstrate that reinforcement modification helps to improve the performance of grouted concrete, with optimal behavior highly dependent on loading mode. CF-reinforced specimens with strong interfacial bonding exhibit the highest compressive strength, which is 150% higher than the bearing capacity of intact concrete, but are prone to brittle fracture under loading involving tension-shear interaction. GF reinforced specimens with moderate interfacial bonding exhibit better load-bearing capacity under tensile-shear stress interaction, reaching a normalized splitting peak load of 0.95 at a grouting thickness of 5 mm. PE-reinforced specimens with weak interfacial bonding provide relatively extensive energy dissipation. A 3 mm grouting layer shows favorable performance among the tested thicknesses, balancing load transfer enhancement and defect control. A single wet–dry cycle temporarily improves performance, possibly due to epoxy post-curing and pore filling, whereas repeated cycling generates cumulative micro-damage. GF- and CF-reinforced systems demonstrate the most stable resistance to short-term wet–dry conditioning. These findings provide guidance for loading-mode-dependent reinforcement selection in epoxy grouting applications.

## 1. Introduction

Concrete, the most widely used artificial building material in the world today, is widely used in bridges, tunnels, dams, and industrial and civil building structures. However, the inherent low tensile strength and high brittleness of concrete materials make them prone to cracking when subjected to multiple coupled effects such as long-term load, temperature changes, shrinkage creep, and freeze–thaw cycles during service [1,2,3,4,5]. The appearance of cracks not only reduces the stiffness and load-bearing capacity of the structure, but also provides a fast track for harmful media such as chloride ions, sulfates, and moisture to invade, accelerating steel corrosion and concrete carbonation, seriously threatening the long-term safety and service life of the structure [6,7,8,9,10]. Crack repair has become one of the key technical challenges that urgently need to be solved in the field of civil engineering [11,12].

Surface sealing, grouting, and filling are the main technical methods in current concrete crack repair engineering [13,14,15,16]. Among them, grouting repair technology, with its advantages of convenient construction, minimal disturbance to the original structure, and controllable repair depth, has become the main process for dealing with internal through cracks and deep microcracks in concrete [17]. Epoxy resin is one of the most ideal polymer materials for structural repair due to its excellent mechanical strength, good chemical stability, and outstanding bonding performance to the concrete matrix [18,19,20,21]. Epoxy resin grouting material can form a strong interface bond with the surface of concrete cracks through mechanical meshing and chemical bonding, effectively restoring the integrity and load-bearing capacity of the structure [22,23,24]. Under the combined action of salt erosion and freeze–thaw cycles, epoxy resin can improve the resistance of concrete to salt erosion and freeze–thaw damage by inhibiting crack propagation and reducing pore development [25]. Zhang et al. [26] compared the durability of ordinary resin and graphene-modified epoxy vinyl resin in an ordinary concrete environment using a 60 °C accelerated aging method. Wang et al. [27] evaluated the application of reduced graphene oxide (rGO) modified epoxy resin composites in improving the durability and corrosion resistance of reinforced concrete beams. Among them, the optimal concentration of 0.2 wt.% rGO significantly improved the corrosion resistance and impermeability of the epoxy coating. However, engineering practice feedback shows that pure epoxy resin grouting materials have inherent defects in crack repair. After curing, epoxy resin has a high crosslink density, limited molecular chain movement, and typical brittle fracture characteristics. This high brittleness makes the repair layer prone to microcracks and brittle fracture at the interface when subjected to subsequent loads or temperature deformations [28]. In addition, the thermal expansion coefficient of epoxy resin is significantly higher than that of concrete, which leads to uncoordinated thermal and shrinkage stresses at the repair interface in harsh environments, which will repeatedly accumulate, resulting in interface debonding failure [23,29].

Fibers, as high-toughness materials, serve as effective means to overcome the inherent brittleness of epoxy resin. Hu et al. [30] prepared fiber-epoxy composites by incorporating silane-modified bamboo fibers into epoxy, and revealed the mechanism by which fibers improve the intrinsic brittleness of epoxy through crack bridging and energy dissipation via fiber pull-out. Zhang et al. [31] fabricated basalt fiber/silicone epoxy composites and achieved matrix toughening by optimizing interfacial bonding. These studies collectively demonstrate that fibers embedded in the resin matrix play crucial roles in bridging cracks, transferring stress, and dissipating fracture energy, thereby significantly enhancing the toughness, crack resistance, and impact resistance of the composites. Pang et al. [32] developed a basalt fiber-reinforced waterborne epoxy-concrete composite repair material (WECM), and confirmed the synergistic toughening effect between waterborne epoxy and fibers, where the epoxy forms a coating layer on the fiber surface to improve the interfacial bonding between fibers and the concrete matrix. Li et al. [33] designed a waterborne epoxy-modified carbon fiber-reinforced cementitious composite for crack repair, and investigated the toughening and strengthening mechanisms imparted by epoxy and carbon fibers to concrete. However, the composite repair materials in both studies are ternary composite systems consisting of fibers, waterborne epoxy, and cementitious matrix, wherein the waterborne epoxy serves as a supplementary modifying component incorporated into the cement-based material, and the toughening effect of fibers is reflected in the overall mechanical enhancement of the bulk composite, rather than in the improvement of the epoxy grout layer itself. Zhang et al. [34] developed a composite overlay using fiber fabric and polyurethane-modified epoxy for repairing fatigue cracks in epoxy asphalt mixture (EAM) pavements on steel bridge decks. The resin thoroughly impregnates the fiber fabric to form a robust interface, and upon loading, interfacial slip between the fibers and resin, along with fiber pull-out, dissipates substantial fracture energy through friction, thereby transforming the pure brittle fracture mode of epoxy asphalt at low temperatures into a shear ductile failure with pronounced yielding. Rousakis et al. [35] employed multi-walled carbon nanotubes to modify three types of epoxy resins to obtain nano-enhanced repair matrices, which were combined with glass fiber fabrics and polypropylene fiber ropes to form a hybrid confinement system; the study confirmed the synergistic toughening and strengthening effects of nano fillers and macro fibers on resins and compressed concrete. Hsu et al. [36] proposed a combined repair and strengthening technique using epoxy grouting and carbon fiber fabric for rehabilitating concrete slabs cracked by reinforcement corrosion. Their study demonstrated that the synergistic interaction between the high tensile properties of carbon fibers and the adhesion of epoxy resin achieves effective toughening and strengthening of the repaired concrete. The common feature of the above studies is that all adopt pre-formed fiber fabrics or ropes, where the toughening effect of fibers is confined to the surface zone or confinement region of the structure.

The above studies demonstrate that fiber-reinforced or otherwise modified epoxy resin composites have shown promising toughening effects at the material level. However, existing research can be broadly categorized into two groups. One group focuses on the mechanical characterization of reinforced resin composites, where the toughening mechanisms of the reinforcing materials have been well validated at the composite level, but few studies have systematically investigated the mechanical behavior of the composite system formed after grouting these materials into concrete. The other group combines epoxy repair systems with fiber fabrics or ropes, achieving structural strengthening through surface bonding or wrapping. In this case, however, the reinforcing effect is confined to the surface zone or confinement region, failing to address the full-section reinforcement of deep-seated internal defects. Moreover, previous studies have shown that fiber-reinforced polymers (FRP) may debond under oblique compression, and even the application of two layers of carbon FRP (CFRP) is insufficient to improve the shear capacity of low-strength concrete [37,38].

This study focuses on the concrete composite system formed after grouting with reinforced epoxy materials. Carbon fiber, glass fiber, and high-density polyethylene microspheres were respectively compounded with epoxy resin to produce three grouting materials with distinct reinforcement morphologies and toughening mechanisms. These materials were injected into the gap between concrete substrates to fabricate a laboratory-scale concrete–epoxy interlayer-concrete composite specimen model with a controlled grout layer thickness, representing an idealized laboratory model of a grout-filled gap between concrete substrates. While real cracks exhibit irregular geometries and non-uniform widths, this model enables systematic investigation of the interfacial failure behavior of reinforced epoxy-grouted concrete under various loading conditions. The selection of the three reinforcement materials is based on their distinctly different elastic moduli and toughening mechanisms, as well as their different interfacial bonding characteristics and bond strengths with the epoxy grout–concrete interface, as established in our previous work [39]. This study aims to elucidate how reinforcement materials with different morphologies and moduli govern the mechanical behavior and interfacial failure modes of epoxy-grouted concrete composite systems, and to provide experimental guidance for the design and material selection of epoxy-based grouting composites for concrete applications.

## 2. Materials and Methods

### 2.1. Materials

#### 2.1.1. Concrete

The cement used in this work is PO42.5 grade ordinary Portland cement, and ordinary river sand is selected as the fine aggregate. The concrete mix ratio is shown in Table 1.

#### 2.1.2. Epoxy Resin and Reinforcing Materials

**Figure 1:**
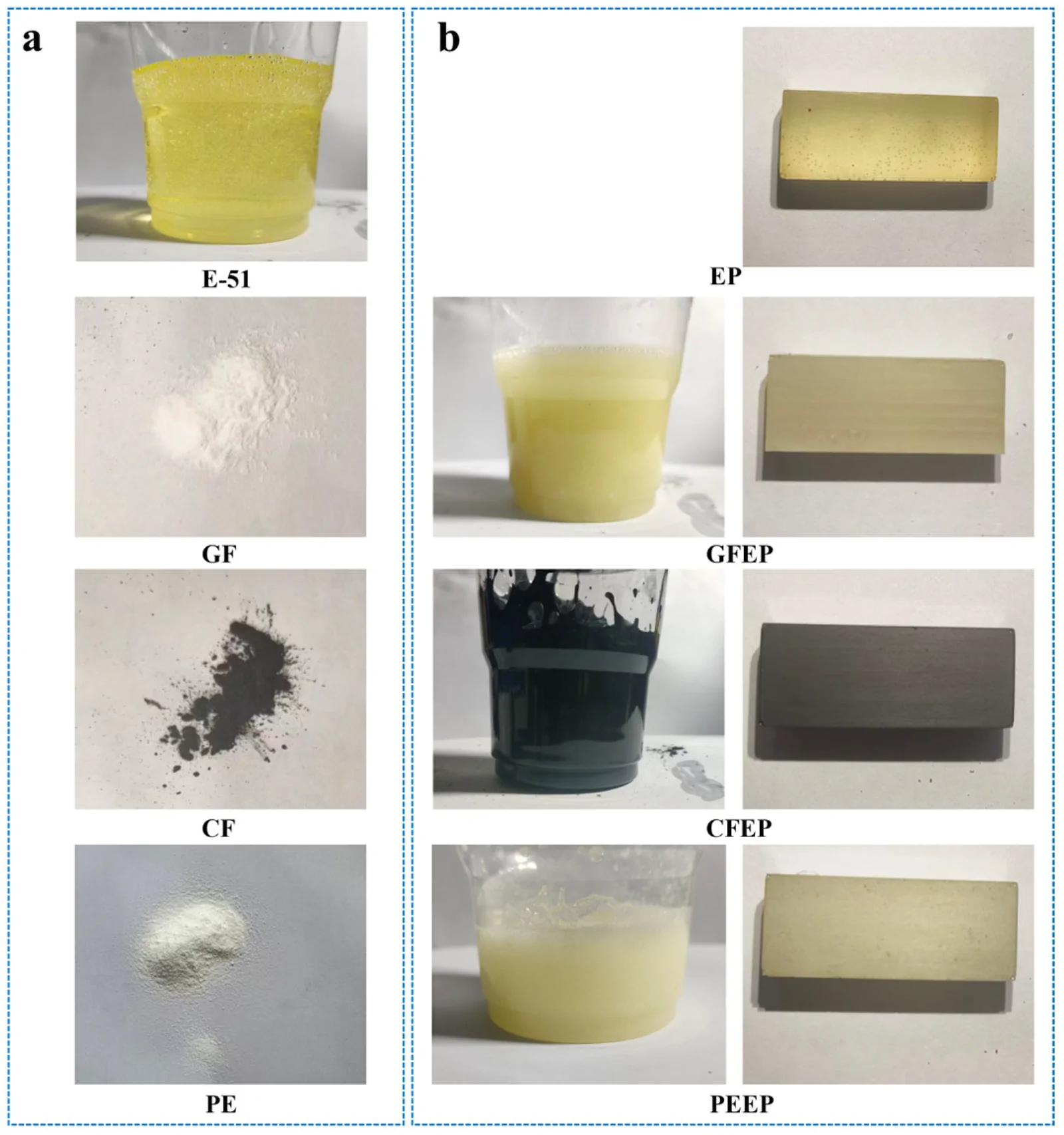
Physical appearance of (**a**) epoxy resin and three types of reinforcing materials, (**b**) the four grouting materials before and after curing (EP, GFEP, CFEP, and PEEP).

Epoxy resin (E-51, bisphenol A-based) and low-temperature curing agent (JH45) are provided by Hangzhou Wuhuigang Adhesive Co., Ltd., Hangzhou, China. The epoxy equivalent of epoxy resin E51 is 185–200 g/eq, and the epoxy value is 0.48–0.54 eq/mg. The tensile strength of the cured resin matrix of E51 and JH45 is 26.79 MPa, and the elongation at break is 0.853%. Typical short-cut PAN-based carbon fibers (CF), E-glass fibers (GF), and ordinary high-density polyethylene microspheres (PE) are used to prepare reinforced resin composites. The morphology and basic physical properties of the three types of reinforcing materials are shown in Figure 1a and Table 2, all provided by the manufacturer. PE was selected in microsphere form rather than as short-cut fibers because commercially available PE fibers are limited to millimeter-scale lengths unsuitable for grouting injection, and its toughening mechanism of matrix yielding and particle debonding caused by stress concentration is also in stark contrast to the fiber bridging and pull-out mechanisms of CF and GF. Sodium hydroxide and silane coupling agent KH-550 are used for cleaning and surface modification of reinforcing materials, respectively. The physical morphology of the four grouting materials before and after solidification is shown in Figure 1b. The selection of these three reinforcing materials follows from their distinctly different mechanical properties and interfacial bonding behaviors with epoxy, as documented in our previous investigation [39]. This diversity enables a systematic comparison of reinforcement mechanisms under different loading modes.

### 2.2. Preparation of Reinforced Resin Composite Materials

In this study, CF, GF, and PE were uniformly mixed into the entire epoxy grouting layer, rather than just sticking fiber cloth on the surface of the specimen. This full-thickness reinforcement design is aimed at deep-penetration crack-grouting engineering scenarios, and its microscopic mechanistic foundation has been verified in our previous shear-interface characterization experiments. The uniform dispersion of reinforcing materials throughout the grouting layer enables toughening mechanisms to act on the entire crack thickness, not just limited to the concrete surface. The preparation scheme of reinforced resin is consistent with our previous works [39]. The reinforcing materials were washed and dried sequentially with sodium hydroxide and deionized water, and stirred continuously for 3 h in a KH550 solution at 70 °C to fully modify the surface of the reinforcements. Then the dried reinforcements were slowly added to the epoxy resin at a stirring speed of 100 rpm, followed by the addition of curing agent JH45, with continued stirring. The mass ratio of epoxy resin to reinforcements and curing agent is 100:10:35. Pure epoxy (EP), glass fiber-reinforced epoxy (GFEP), carbon fiber-reinforced epoxy (CFEP), and polyethylene-reinforced epoxy (PEEP) were prepared following the identical preparation procedure described above. As shown in Table 2, CF, GF, and PE differ significantly in morphology, dimensions, aspect ratio, and density. It should be noted that an equal mass fraction (10%) of reinforcement was used for all modified epoxy grout systems in this study. Due to the variation in density among the three materials, the corresponding volume fractions are inconsistent across different groups: approximately 6.2% for CF, 4.3% for GF, and 11.1% for PE. Furthermore, the number of reinforcement entities per unit volume varies considerably due to differences in diameter. Moreover, the discrepancy in reinforcement geometry inevitably affects the crack resistance and toughening performance. Therefore, the differences in mechanical behavior of the grouted specimens reflect the combined effects of the intrinsic material properties of the reinforcements and their geometric characteristics.

### 2.3. Preparation of Concrete and the Grouted Concrete

#### 2.3.1. Compression Test

**Figure 2:**
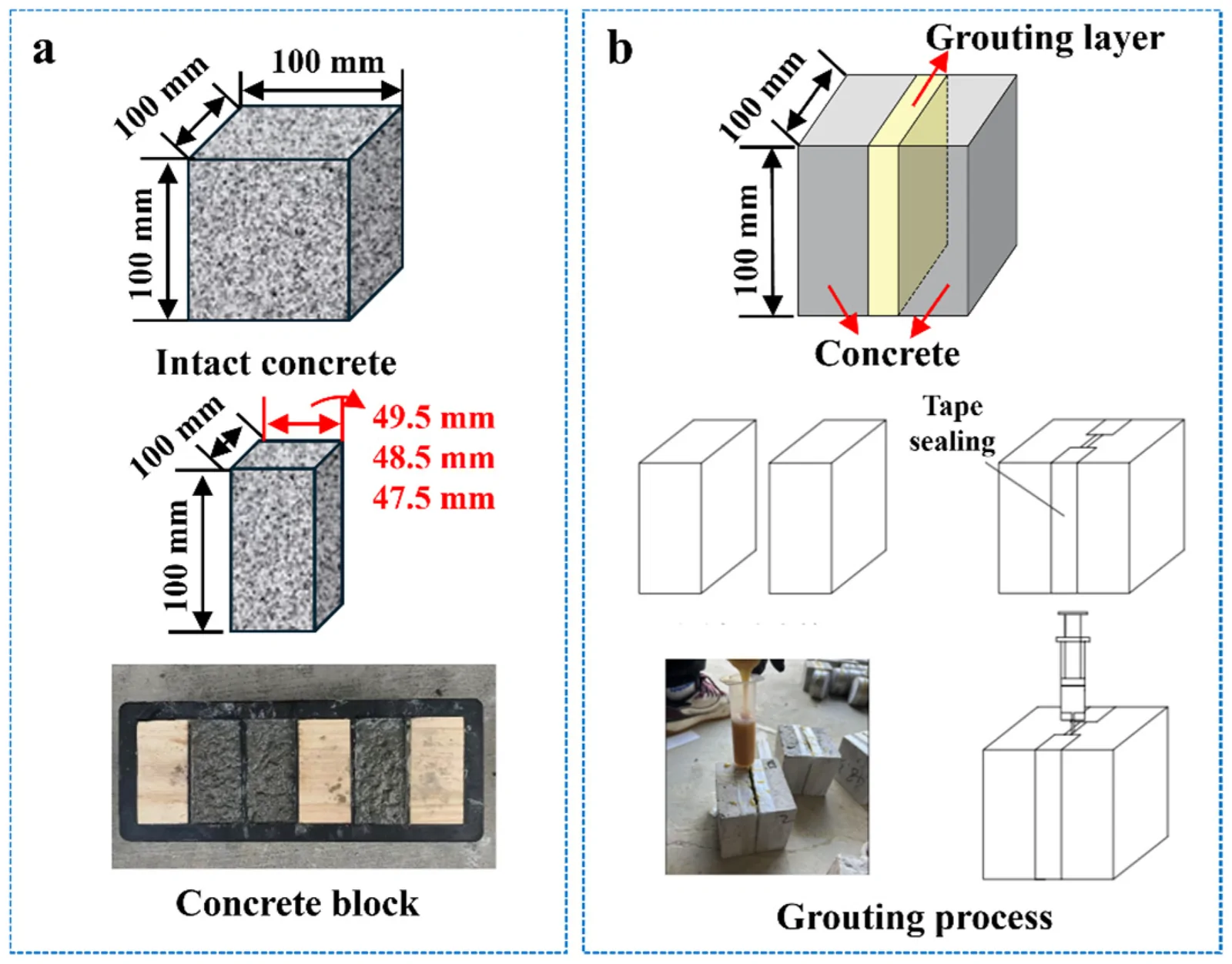
Schematic diagram of specimen preparation for compression test. (**a**) Concrete specimens; (**b**) grouted concrete specimens.

Standard cubic molds with dimensions of 100 mm × 100 mm × 100 mm and custom birch spacers were adopted to fabricate intact concrete blocks and two-part concrete blocks with a controlled gap of specific thicknesses (Figure 2a). It should be emphasized that the concrete substrate and the epoxy grout were not cast continuously. Instead, the concrete specimens were first cast and subjected to a full 28-day standard curing period to ensure complete hydration and strength development. For grouted concrete specimens, grouting interlayer thicknesses of 1 mm, 3 mm, and 5 mm were designed. While real cracks exhibit irregular geometries and variable widths, the use of controlled uniform-thickness layers in this laboratory study enables systematic isolation of the effects of fiber type, grouting thickness, and loading mode on the interfacial behavior of fiber-reinforced epoxy grouts with concrete. After curing, remove the concrete block from the curing tank, dry it at room temperature, and carefully prepare its bonding surface according to standardized cleaning procedures, including cleaning the dust, slurry, and loose particles on the surface to be bonded, and then wiping it with anhydrous ethanol to eliminate residual trace pollutants. The surfaces were then allowed to dry completely at room temperature before grouting. As illustrated in Figure 2b, the dried and surface-cleaned concrete blocks were sealed along their edges to form a leak-proof fixed cavity with a reserved grouting inlet. The prepared grouting materials were then injected into the cavity to fabricate EP-grouted concrete (EPC), GFEP-grouted concrete (GFEPC), CFEP-grouted concrete (CFEPC), and PEEP-grouted concrete (PEEPC), respectively. This two-stage fabrication protocol ensures that the measured performance reflects the actual bonding behavior between the matured concrete substrate and the subsequently applied epoxy grout, rather than the mechanical interlocking developed during co-casting or early-age contact. It is worth emphasizing that, although epoxy grouting is typically applied to fine cracks, the grouting layer thickness varies widely in practice. For cracks below 2 mm, the resin alone provides sufficient penetration, and the micrometer-scale reinforcing materials used here are fully compatible. Meanwhile, practical repair often involves V- or U-shaped grooving for structural cracks, while shallow spalling and honeycomb defects require thicker adhesive layers. Accordingly, building on our previous work, grouting thicknesses of 1, 3, and 5 mm were selected to represent a range of grout layer conditions, from narrow gaps to widened grooves and shallow surface defects.

#### 2.3.2. Three-Point Bending Test

**Figure 3:**
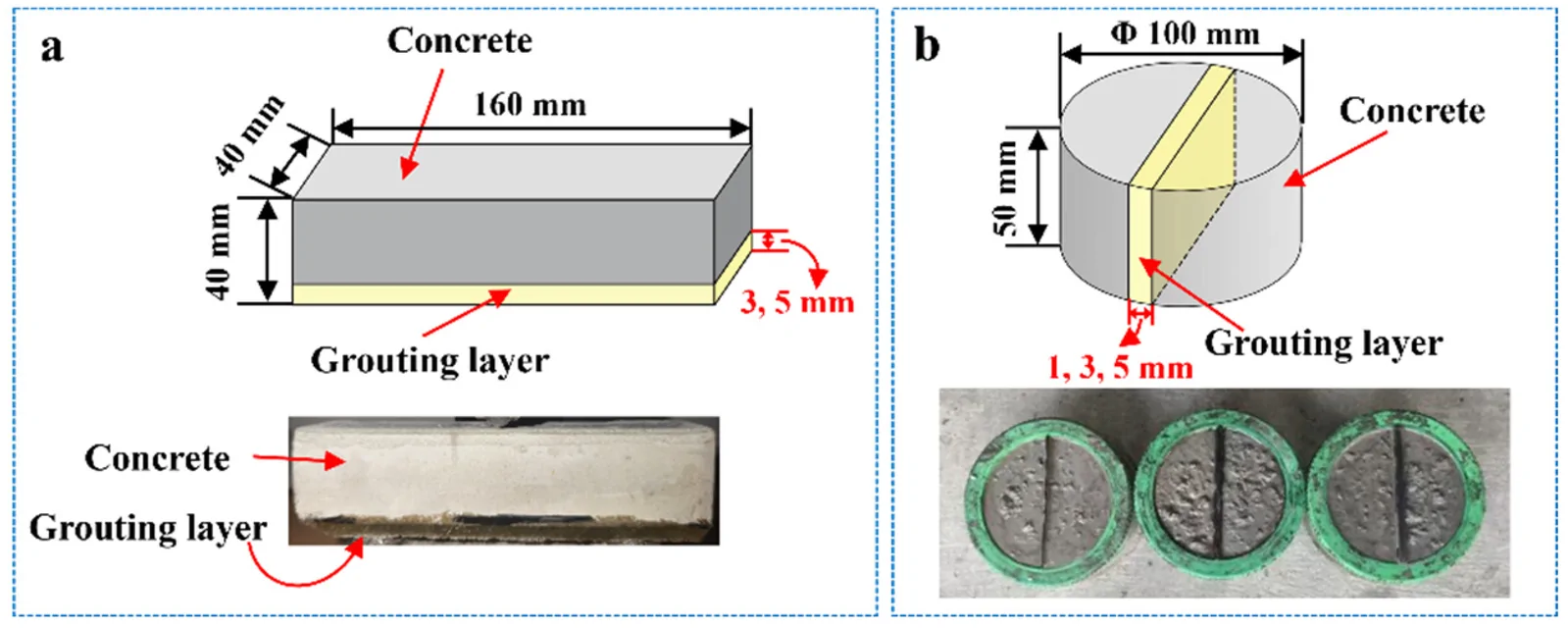
The specimens of (**a**) three-point bending test; (**b**) Brazilian splitting test.

Concrete specimens with dimensions of 160 mm × 40 mm × 40 mm were prepared for three-point bending tests. Under bending load, the bottom region of the specimens is dominated by tensile stress. To characterize the surface cracking behavior of flexural members, grouted concrete specimens adopted a two-layer configuration, as presented in Figure 3a. Grouting layer thicknesses of 3 mm and 5 mm were designed, and the resin grout layer was arranged at the tensile bottom of each specimen. The fabrication, curing, and grouting procedures of concrete specimens were consistent with those adopted for compression tests, and four types of grouted concrete specimens, namely EPC, GFEPC, CFEPC, and PEEPC, were prepared accordingly.

#### 2.3.3. Brazilian Splitting Test

Similar to the compression test, the Brazilian splitting test was adopted to characterize the interfacial performance of specimens with penetrating internal cracks. Cylindrical specimens with dimensions of Φ100 mm × 50 mm were prepared, and the grouted concrete specimens possessed a three-layer structure consisting of concrete matrix, resin grout interlayer, and concrete matrix. As illustrated in Figure 3b, the resin grout interlayer was arranged along the central axis of each cylindrical specimen, with grout thicknesses of 1 mm, 3 mm, and 5 mm designed in this study. The fabrication, curing, and grouting procedures of concrete specimens were identical to those used for compression tests.

#### 2.3.4. Wet–Dry Cyclic Treatment

To evaluate the effects of wet–dry cycles on the interfacial stability and mechanical degradation of grouted concrete, accelerated wet–dry cyclic aging treatments were carried out on intact concrete and four types of grouted concrete (EPC, GFEPC, CFEPC, and PEEPC). One complete wet–dry cycle was defined as the procedure in which specimens were fully immersed in tap water for 24 h, followed by oven-drying at 60 °C for 24 h. Specimens designated for compression and three-point bending tests underwent 0, 1, 3, or 5 wet–dry cycles. After aging, all specimens were conditioned at 20 °C and 60% relative humidity for 24 h to minimize internal temperature and moisture gradients before testing. It should be noted that the wet–dry cycling program in this study is designed as a short-term wet–dry conditioning test rather than a comprehensive long-term durability assessment. The primary purpose is to reveal the early-stage degradation trends and mechanisms of different reinforced epoxy systems under cyclic moisture exposure, rather than to predict long-term service life. The selected cycle levels were chosen to capture the transition from initial post-curing enhancement to the onset of cumulative damage, providing mechanistic insights into the competition between toughening and degradation.

### 2.4. Test Procedure

**Figure 4:**
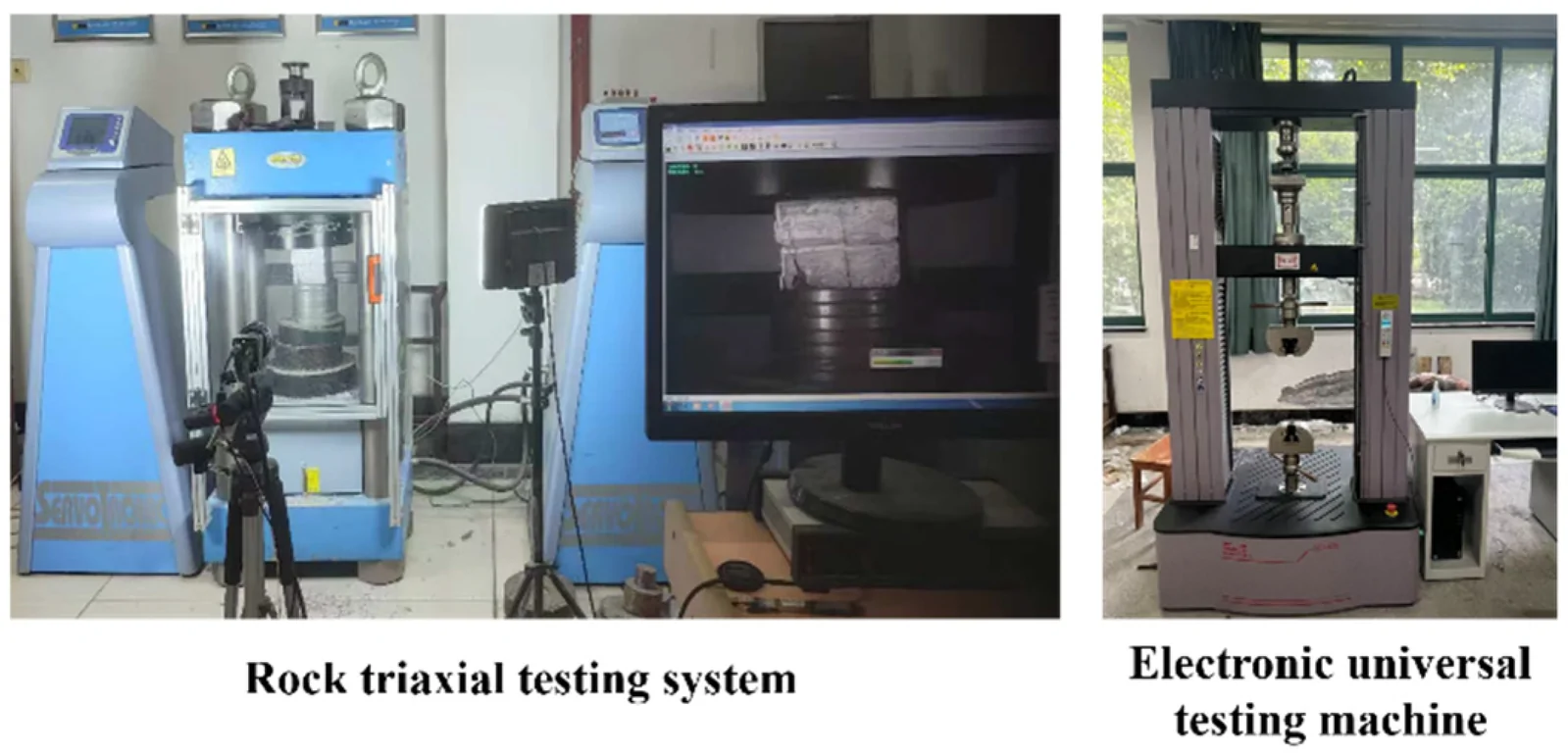
Schematic diagram of test apparatus.

Compression tests were performed using a rock triaxial testing system (C89-19CR) purchased from Shanghai Zhuo Zhi Li Tian Technology Development Co., Ltd., Shanghai, China, while three-point bending and Brazilian splitting tests were conducted using an electronic universal testing machine (WANCE WDW-100A) purchased from Shenzhen Wance Testing Equipment Co., Ltd, Shenzhen, China (Figure 4). Both testing systems provide load and displacement measurements with an accuracy of ±0.5% of the indicated value. The displacement acquisition resolution of the electronic universal testing machine is 0.0001 mm. The loading protocols for each test were as follows.

For the compression test, a load-controlled loading mode was adopted at a rate of 0.5 MPa/s, following the standard procedure for concrete cube compression testing. The test was terminated when the applied load dropped below 90% of the peak load or when the specimen exhibited complete structural collapse. Rigid steel loading platens with a diameter of 150 mm were used, which completely cover the surface area of the specimen.

For the three-point bending test, a displacement-controlled loading mode was applied at a rate of 0.5 mm/min, consistent with the quasi-static loading regime recommended for flexural testing of concrete materials. The test was terminated when the applied load dropped below 10% of the peak load or when the specimen lost its load-bearing capacity. The support span was 100 mm, with cylindrical steel supports of 10 mm in diameter. Displacement was measured by the machine crosshead transducer, including both specimen deflection and machine compliance. The machine contribution is consistent and minor across all specimens under identical conditions, thus not affecting relative comparisons among different specimens.

For the Brazilian splitting test, a displacement-controlled loading mode was adopted at a rate of 0.4 mm/min. The test was terminated when the applied load dropped to a level below 10% of the peak load or when the specimen experienced complete fracture. A wooden plywood with dimensions of 50 mm × 15 mm × 4 mm was placed between the loading platens and the cylindrical specimen along the loading axis to distribute the compressive load and prevent localized stress concentrations at the contact points. The displacement measurement in the Brazilian splitting test is consistent with the bending test, measured by the machine crosshead transducer of WANCE WDW-100A, including both specimen deflection and machine compliance.

Digital image correlation (DIC) was employed to capture surface strain distribution and damage evolution. All specimens were coated with speckle patterns 24 h before testing. During loading, a PIV camera (Beijing Cube Heaven Technology Co., Ltd., Beijing, China, SM-5M200) equipped with MicroVec software (V5.1) was positioned perpendicular to the speckled surface to record speckle images at a frame rate of 14 frames per second (fps) with a resolution of 2456 × 2058 pixels. The recorded images were subsequently processed using the open-source MATLAB-based DIC software (v1.2.2) Ncorr to compute full-field displacement and strain fields. In the Ncorr analysis, a subset radius of 48 pixels and a subset spacing of 5 pixels were adopted, achieving a displacement measurement accuracy of approximately 0.01 pixels and a strain resolution of approximately 200 με, which is sufficient for the strain levels observed in this study. All tests were conducted on three replicate specimens per configuration to ensure statistical validity.

The mechanical parameters, including compressive strength (*f_cu_*, MPa), were calculated following Equation (1).

(1) $$f_{cu}=\frac{P_{max}}{A}$$

where *P_max_* is the ultimate failure load (N), and *A* is the compressive cross-sectional area of the cube (mm^2^).

The strength ratio (*η*) was defined to represent the bearing capacity of grouted concrete relative to intact concrete, as calculated in Equation (2).

(2) $$\eta =\frac{f_{grouted}}{f_{concrete}}\times 100\%$$

where *f_grouted_* and *f_concrete_* are the strength of the grouted specimen and the intact concrete specimen under the same wet–dry cycle, same thickness of grouting layer, and same test type, respectively.

The flexural and splitting tensile tests were conducted with reference to standard methods for concrete specimens [40,41]. Equations (3) and (4), which are derived based on the elastic stress field of homogeneous and isotropic materials, were adopted to determine the flexural strength (*f_b_*, MPa) and splitting tensile strength (*f_t_*, MPa), respectively, of the intact concrete specimens.

(3) $$f_{b}=\frac{3P_{b,max}L}{2bh^{2}}$$

where *P_b_*, max is the bending ultimate peak load (N), *L* is the support span (mm), *b* and *h* are the cross-sectional width and height of the specimen (mm), respectively. In this work, *L* is 100 mm.

(4) $$f_{t}=\frac{2P_{t,max}}{\pi Dt}$$

where *P_t_*_,*max*_ is the ultimate failure load of the Brazilian test (N), *D* and *t* are the diameter and thickness of the specimen (mm), respectively.

However, the grouted concrete specimens are heterogeneous composite structures with distinctly different elastic moduli between the concrete and the grouting layer, making Equations (3) and (4) inapplicable. Accordingly, the peak load is used directly in this study to characterize the flexural and tensile load-bearing capacities of the grouted specimens, consistent with established practice in structural repair literature [42,43].

## 3. Results and Discussion

### 3.1. Compressive Behavior of Grouted Concrete

**Figure 5:**
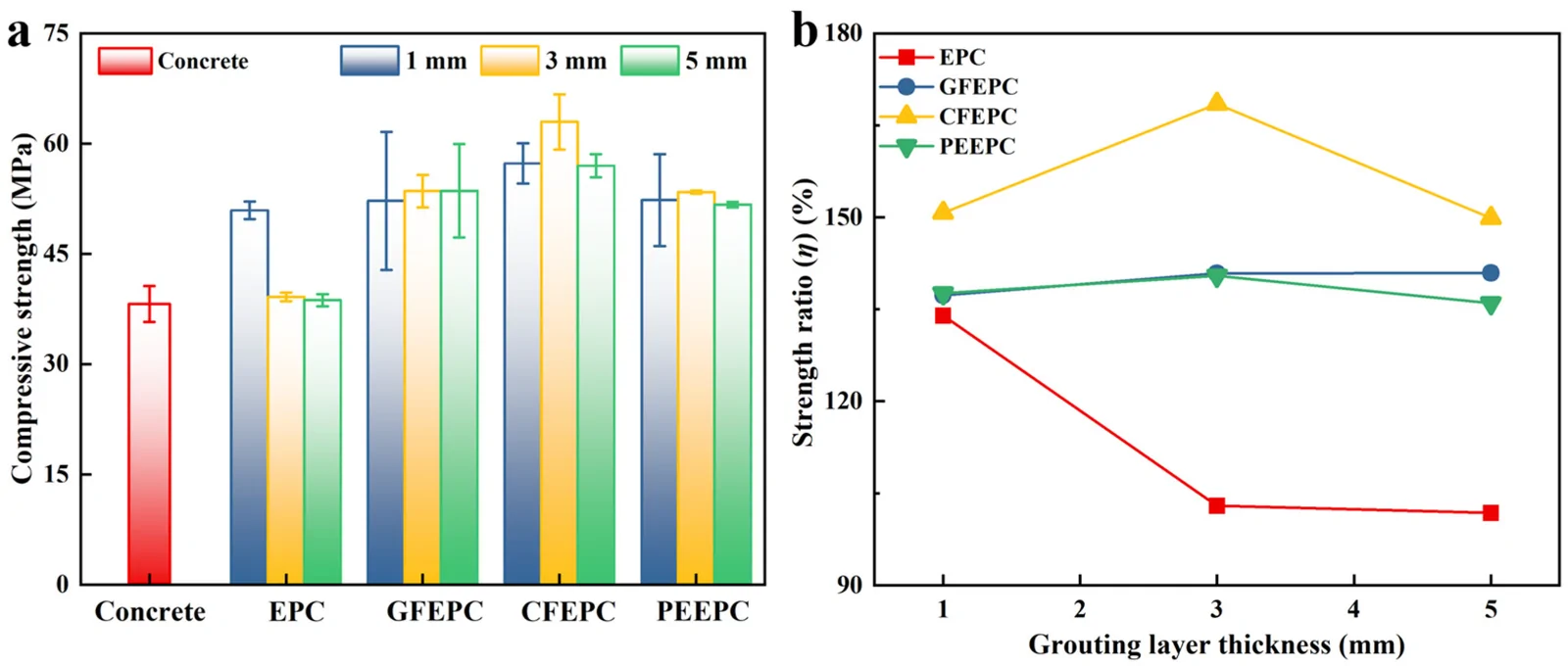
Compressive performance of grouted concrete with different grouting layer thicknesses. (**a**) Compressive strength; (**b**) bearing capacity relative to intact concrete.

Figure 5 presents the compressive strength and corresponding recovery rate of four types of grouted concrete at different grout layer thicknesses. All grouted specimens exhibit higher compressive strength than intact concrete, and the compression bearing capacity is governed by both grouting layer thickness and fiber type. At a grouting thickness of 1 mm, the compressive bearing capacity of EPC is 134% of that of intact concrete. However, as the grouting thickness increases, the compressive strength declines considerably and approaches that of intact concrete. This reduction is attributed to the increase in inherent micropores within the epoxy matrix with increasing grouting thickness. The reinforced grouting systems substantially overcome the deficiencies of the epoxy matrix. Among them, the bearing capacity of CFEPC is over 150% of that of intact concrete, and it first increases and then decreases with the increase in grouting thickness, reaching a peak at 3 mm. GFEPC and PEEPC also exhibited stable compressive performance at all three grouting thicknesses, reaching approximately 140% of the intact concrete bearing capacity.

**Figure 6:**
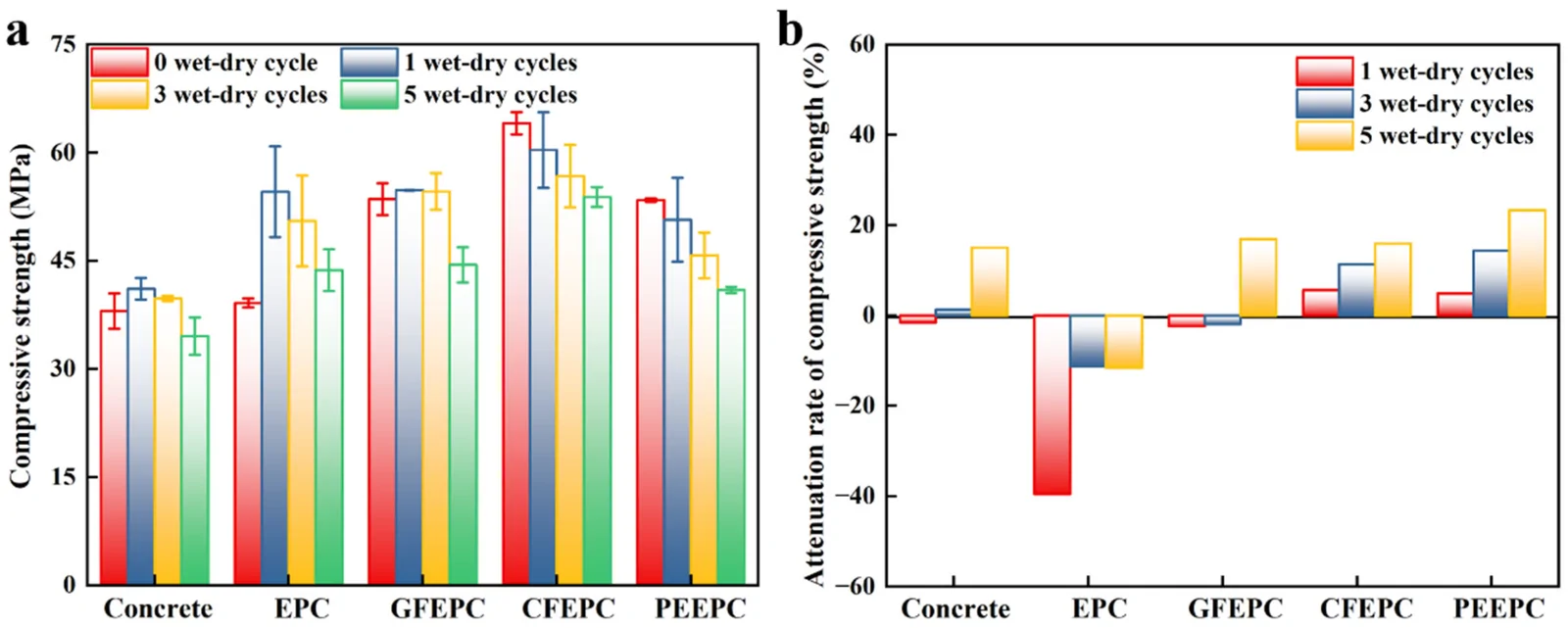
Compressive performance of grouted concrete subjected to wet–dry cycles. (**a**) Residual compressive strength; (**b**) attenuation rate of compressive strength after wet–dry cycles.

The compressive strength of intact concrete and grouted concrete with a 3 mm grouting layer thickness after wet–dry cycles is shown in Figure 6. The compressive strength of intact concrete exhibits minor fluctuations under a limited number of wet–dry cycles, which is attributed to the continuous hydration of the cementitious matrix, where surface pores are densified by hydration products. However, after five repeated wet–dry cycles, damage to the internal capillary pores leads to a strength reduction of over 15%. In contrast, after one wet–dry cycle, the epoxy-grouted EPC specimens show a notable increase in compressive strength, with an enhancement rate exceeding 39%. The compressive strength of GFEPC is also slightly higher than that of the uncycled specimens. When the cycle number increases to three and five, the compressive strength of almost all specimens degrades, yet remains higher than that of intact concrete. No matter how many cycles, CFEPC consistently maintains the highest compressive strength. During a single wet–dry cycle, a small amount of moisture may permeate into the microscopic pores within the epoxy matrix, potentially triggering further crosslinking of the epoxy resin and enhancing the density of the matrix. Meanwhile, micropores at the concrete-resin interface may be partially filled with hydration products, possibly reducing interfacial defects under compression. These effects could explain the short-term increase in compressive strength of EPC after one cycle. As the number of wet–dry cycles increases, the pores undergo repeated swelling upon water absorption and shrinkage upon drying. The resulting cyclic stresses generate throughgoing microcracks, leading to a decline in compressive load-bearing capacity. It is worth mentioning that the observed degradation after five cycles should be interpreted as an early-stage indication of the material’s susceptibility to short-term cyclic moisture exposure, rather than a prediction of long-term durability.

**Figure 7:**
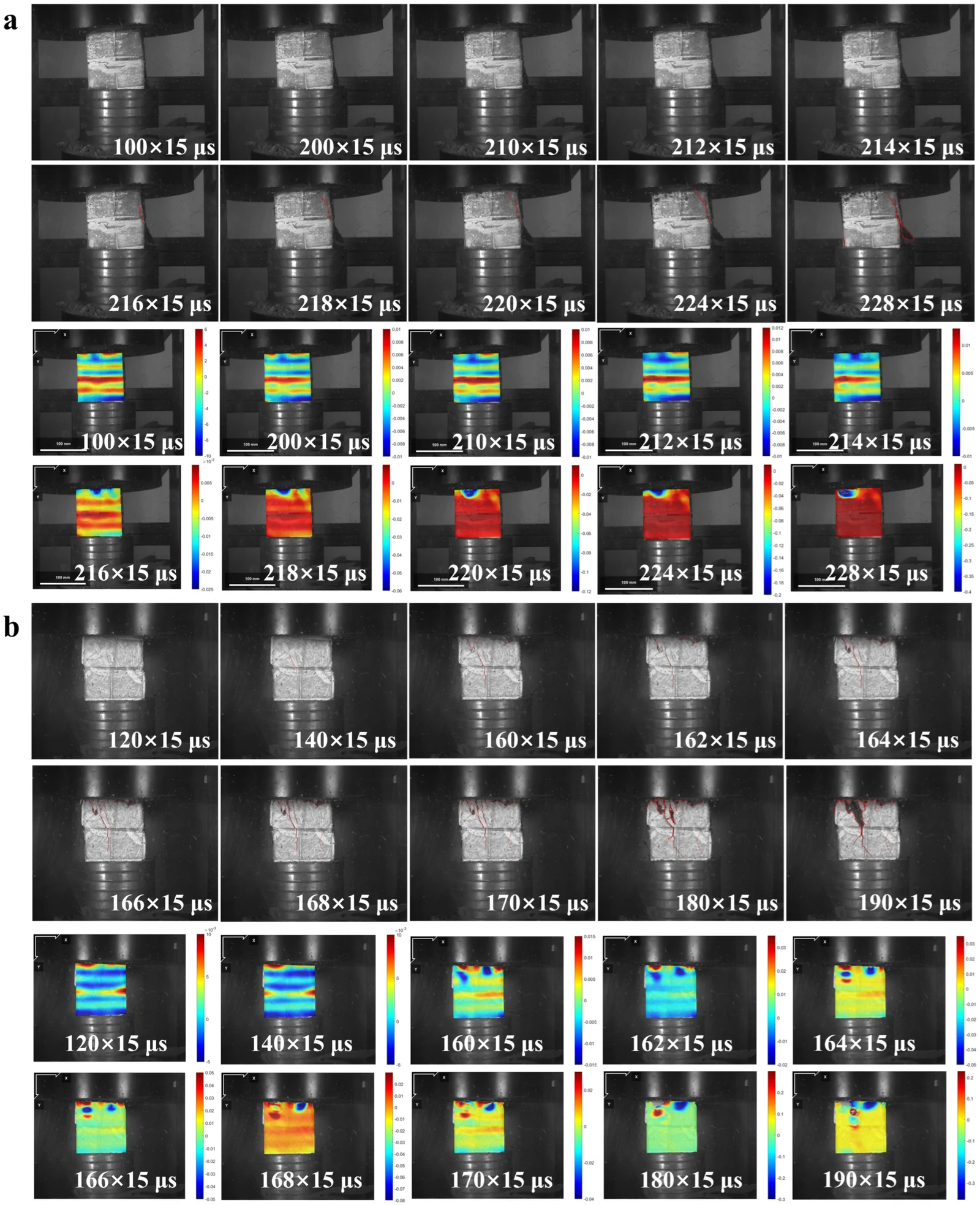
DIC images of compression tests on specimens without wet–dry cycles. (**a**) EPC; (**b**) CFEPC.

Figure 7 presents time-series surface images and vertical compressive strain maps of EPC and CFEPC specimens with a 3 mm grouting interlayer during uniaxial compression. At the early loading stage, both EPC and CFEPC undergo uniform elastic compressive deformation, with vertical compressive strain distributing evenly in horizontal layers along the specimen height, and no apparent localized damage is observed on either surface. As loading proceeds, distinct differences emerge in the onset and evolution of strain localization between the two grouted systems. For the EPC specimen, the strain magnitude remains within ±0.012 up to frame 214, with only inherent interfacial gaps between concrete and epoxy visible on the surface. At frame 216, however, a localized high-amplitude compressive strain concentration zone suddenly forms at the top of the specimen, accompanied by the initiation of surface microcracks. This abrupt strain localization indicates that, in the absence of fiber reinforcement, the neat epoxy layer cannot effectively redistribute localized stresses once damage initiates. In contrast, for the CFEPC specimen, scattered multi-point high-strain zones emerge at the corners as early as frame 160, also indicating the initiation of surface microcracks. The earlier yet more dispersed appearance of high-strain zones in CFEPC suggests that carbon fibers begin to engage in stress transfer at a relatively low load level, redistributing the local compressive stresses over a wider area and preventing the formation of a single dominant strain localization band. The most significant discrepancy between EPC and CFEPC lies in their post-peak failure behavior. After the peak load, the high compressive strain zones of EPC abruptly localize and intensify, accompanied by a sharp surge in strain amplitude from 0.02 to 0.4 within eight frames. Interfacial damage rapidly coalesces and penetrates through the specimen, leading to a steep drop in bearing capacity and catastrophic brittle compressive failure without any transitional stage. This behavior is characteristic of the inherent brittleness of the neat epoxy matrix, where once the load-bearing capacity is exceeded, cracks propagate unstably along the weakest path. In CFEPC, however, after the peak load, the high-strain regions expand gradually and uniformly rather than localizing abruptly. As shown in Figure 7b, the surface cracks of the CFEPC specimen gradually disperse and develop across multiple locations between frames 162 and 190, and only gradually penetrate through the specimen, whereas the cracks in the EPC specimen rapidly localize and propagate through a single dominant path immediately after formation. These DIC observations collectively demonstrate that carbon fibers fundamentally alter the compressive failure mechanism of the grouted concrete. CF has a high modulus and can disperse stress concentration at the interface between epoxy resin and concrete, delaying the onset of interface strain localization, as demonstrated by the dispersed multi-point strain concentration at frame 160. On the other hand, under sustained compressive stress, the high-strain region of CFEPC gradually expands rather than mutates, confirming that the gradual debonding and energy dissipation of CF in the resin can suppress the rapid coalescence of damage after peak load. These combined effects transform the narrow and concentrated high-strain band characteristics of EPC into dispersed multiple high-strain zones, alleviating the brittle compression failure of EPC and obtaining the high compressive strength of grouted concrete, as shown in Figure 5.

### 3.2. Flexural Performance and Toughness Enhancement of Reinforced Epoxy-Grouted Concrete

The flexural tests in this study were designed to evaluate the toughening efficiency of reinforced epoxy layers under flexural loading, rather than to simulate actual crack geometries. The resin layer was placed at the tensile bottom to maximize the contribution of the reinforcements to the flexural response, offering a clear basis for comparing toughening mechanisms among different reinforcement systems. Flexural tests were conducted at 0, 3, and 5 wet–dry cycles. The 1 wet–dry cycle condition, which was included in the compression tests to capture the transient post-curing effect, was omitted from the flexural test program to allow a focus on studying the degradation trend over a longer period of time when the sample capacity is limited.

**Figure 8:**
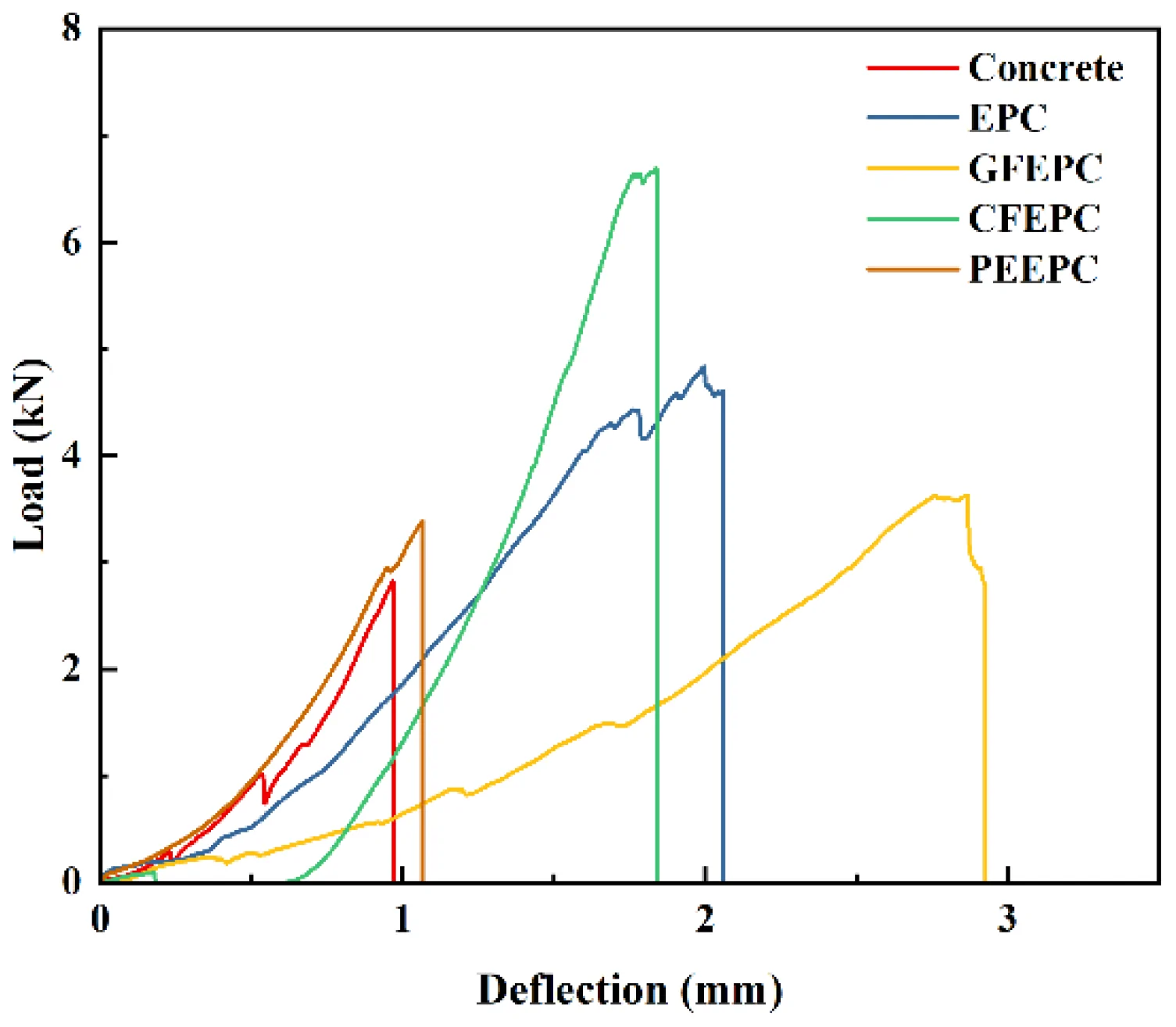
Representative load–displacement curves of intact concrete and grouted concrete specimens with a 3 mm grouting layer (0 wet–dry cycles).

Figure 8 presents representative load–displacement curves for each material with a 3 mm grouting layer (without wet–dry cycling), selected from three replicates as the curve closest to the mean peak load and displacement. All reported quantitative data are averages of three replicates. All specimens exhibit a linear-elastic ascending branch at low displacement levels. Intact concrete exhibits the shortest elastic deformation range; its peak-load displacement is 0.97 mm, followed by an immediate drop of load, reflecting the lowest load capacity and typical brittle failure behavior. Resin grouting improves both the peak load and peak-load displacement of concrete. For EPC, the peak load and peak-load displacement increase by 70% and 106%, respectively, compared with intact concrete. This enhancement is attributed to the ability of epoxy resin to fill interfacial pores and suppress the rapid coalescence of primary cracks. Among the three reinforced systems, CFEPC achieves the highest peak load of 6.7 kN, with a slightly smaller peak-load displacement than EPC. By virtue of bridging action, the high-modulus carbon fibers carry tensile stresses within the flexural tension zone, resulting in improved load-bearing performance. Additionally, CFEPC exhibits the steepest linear-elastic slope, indicating pronounced stiffness enhancement due to carbon fiber incorporation, which leads to abrupt fracture upon reaching the peak load. GFEPC exhibits a peak displacement of approximately 3 mm, with the lowest load growth rate with respect to displacement and the smallest elastic slope. Since glass fiber has a lower elastic modulus than carbon fiber, it provides limited stiffness reinforcement and allows greater deformation accumulation before crack initiation. In contrast, the PEs, owing to their low modulus and weak interfacial bonding with the epoxy matrix, act as stress concentrators that trigger localized matrix yielding and particle debonding, which enhances overall deformation capacity but leads to a relatively lower peak load compared with the fiber-reinforced systems.

**Figure 9:**
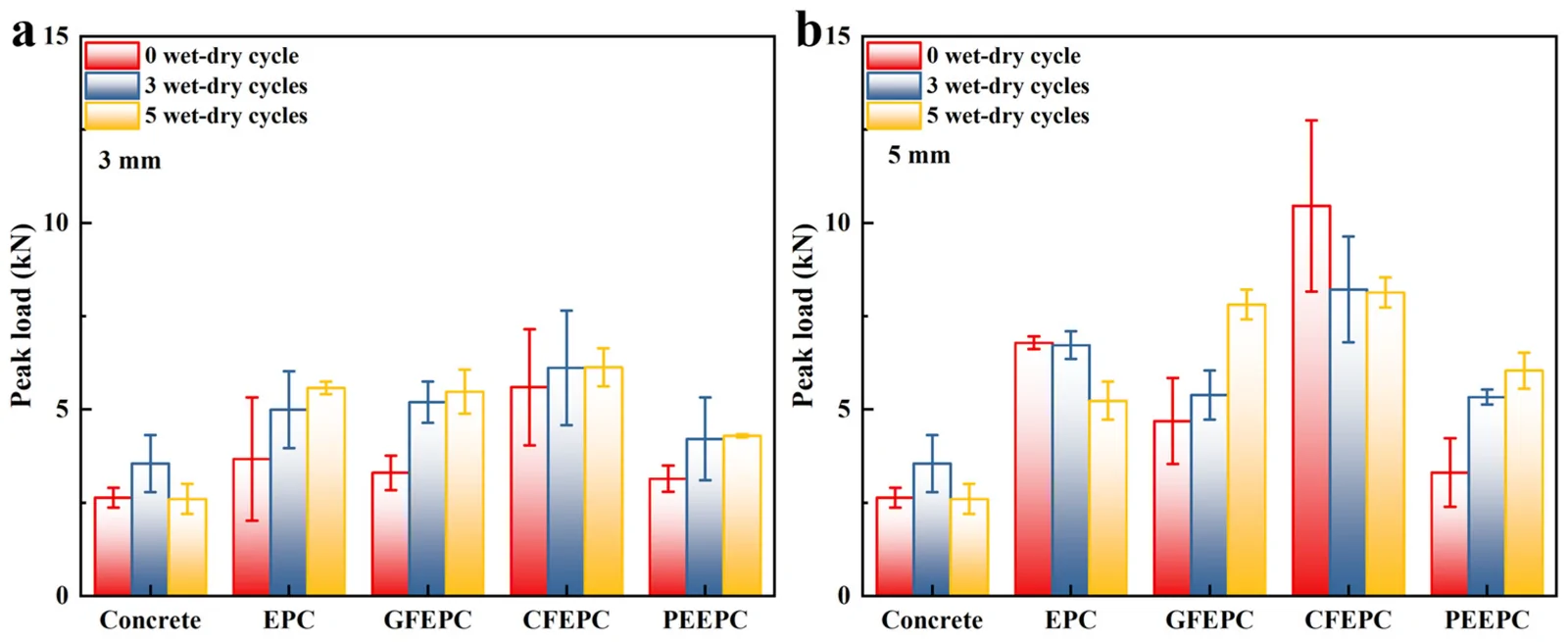
Peak load of different grouted concrete specimens. (**a**) Grouting layer thickness of 3 mm; (**b**) grouting layer thickness of 5 mm.

The peak load of grouted concrete specimens after various numbers of wet–dry cycles is shown in Figure 9. Resin-grouted specimens tend to exhibit higher flexural bearing capacity than intact concrete. Without wet–dry exposure, the peak load of EPC specimens with 3 mm and 5 mm grouting layers shows an increase of 39% and 157%, respectively, compared with intact concrete. As the number of wet–dry cycles increases, the peak load of EPC with a 3 mm grouting layer shows a slight increase, whereas that of the 5 mm EPC specimen declines from 6.78 kN to 5.23 kN after five cycles. The reinforced systems exhibit varying trends with wet–dry cycling. The average peak load of CFEPC reached its highest at both thickness levels, indicating its high bending capacity. Although its peak load degrades considerably with increasing wet–dry cycles at the 5 mm thickness, it remains higher than those of the other grouted systems. In contrast, GFEPC and PEEPC exhibit relatively low peak load before cyclic exposure, but their peak load increases progressively with the number of wet–dry cycles. For EPC, which consists of an unreinforced epoxy matrix, abundant inherent microdefects exist. Prolonged wet–dry erosion promotes interconnection and expansion of internal micropores. In CFEPC, carbon fibers form strong interfacial bonding with the epoxy matrix after surface treatment, leading to severe stress concentration at the fiber-matrix interface. Repeated water infiltration induces cyclic internal stresses due to hygroscopic expansion and dehydration shrinkage, and the accumulated stress initiates interfacial micro-debonding, thereby reducing its flexural bearing capacity. By contrast, GFEPC and PEEPC exhibit moderate to relatively weak interfacial bonding, which enables stress relief through controlled interfacial slip, thereby mitigating internal stresses induced by cyclic swelling and shrinkage.

**Figure 10:**
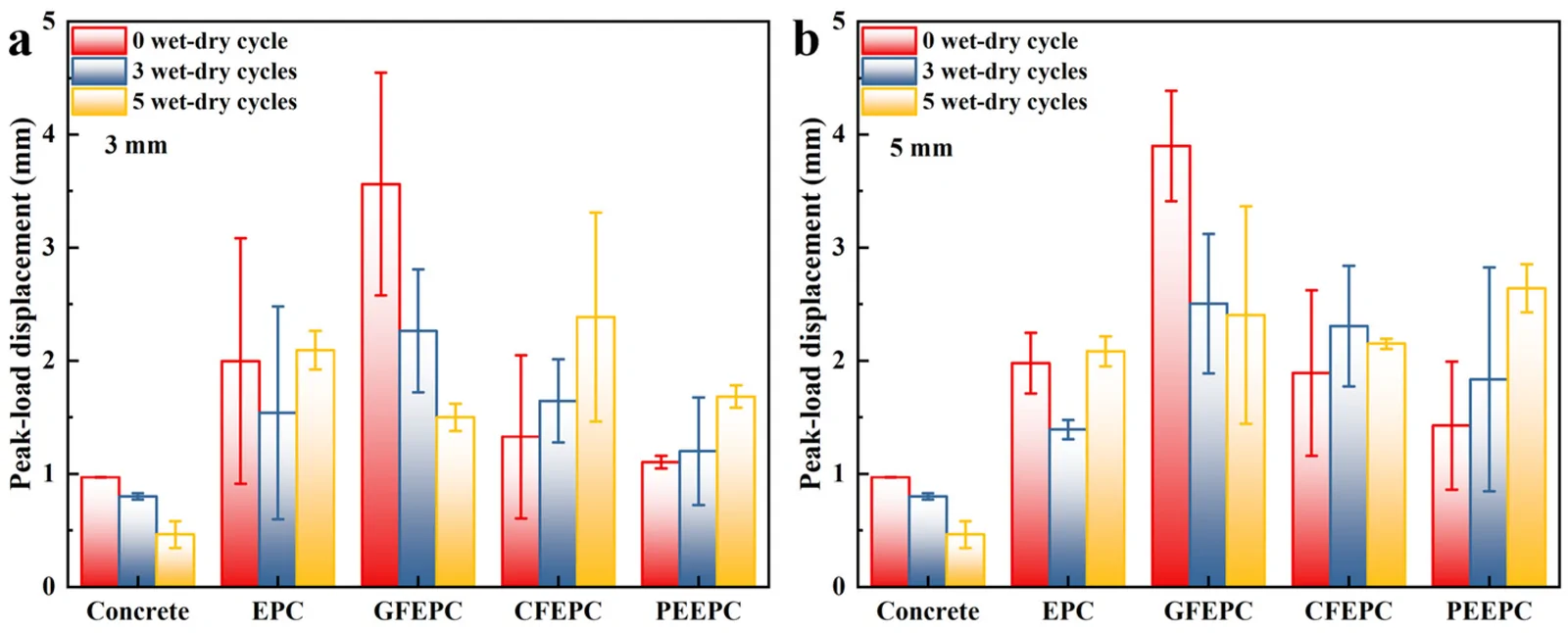
Displacement at peak load of different grouted concrete specimens. (**a**) Grouting layer thickness of 3 mm; (**b**) grouting layer thickness of 5 mm.

For the three-point bending test, the load and displacement data were recorded by the testing machine. The displacement was measured by the machine crosshead displacement transducer of WANCE WDW-100A. Thus, the reported displacement values include contributions from both specimen deflection and machine compliance. All reported quantitative comparisons are based on these recorded values as comparative indices. The machine contribution is consistent and minor across all specimens under identical conditions, thus not affecting relative comparisons. Figure 10 illustrates the displacement at peak load of intact concrete and various grouted specimens after different wet–dry cycles. Intact concrete is intrinsically brittle, allowing pre-existing cracks to propagate and coalesce rapidly. Wet–dry cycling induces pore structure deterioration in concrete, amplifying its brittleness and causing a progressive reduction in displacement at peak load from 0.97 mm at zero cycles to 0.46 mm after five cycles. Under identical testing conditions, the four grouted specimens, EPC, GFEPC, CFEPC, and PEEPC, tend to exhibit higher displacement at peak load than intact concrete. For instance, at zero wet–dry cycles with a 3 mm grouting thickness, the peak displacements of EPC, GFEPC, CFEPC, and PEEPC reach 2.00 mm, 3.56 mm, 1.32 mm, and 1.10 mm, respectively. Even after multiple wet–dry cycles, the mean peak displacements of the grouted specimens remain higher than those of intact concrete under the vast majority of test conditions. When the grouting thickness increases from 3 mm to 5 mm, the displacement at peak load increases for most of the grouted specimens. Among the four grouted systems, GFEPC generally exhibits the largest displacement under most conditions, indicating its outstanding deformation capacity. Reinforcement type and grouting thickness jointly govern the response of peak displacement to wet–dry cycling. Our previous work, based on SEM and nanoindentation characterization, has demonstrated that CF-reinforced CFEP exhibits the strongest interfacial bonding with concrete, followed by GFEP, while PEEP shows the weakest interfacial bonding with concrete [39]. This difference in interfacial characteristics provides a basis for understanding the distinct behaviors of the three reinforced systems under wet–dry cycling. For GFEPC, the peak displacement after five wet–dry cycles at a grouting thickness of 3 mm is only 1.50 mm, which is considerably lower than that of GFEPC specimens under other conditions and also lower than those of the other three grouted systems under the same conditions. This suggests that in the thin grouting layer, the interface between GFEP and concrete may undergo hydrolytic degradation during repeated wet–dry alternation, which could lead to a loss of fiber bridging efficiency and promote crack propagation along the weakened interface. At a thickness of 5 mm, however, the moisture penetration path is prolonged, leaving a well-preserved core zone within the grouting layer, and the attenuation trend is substantially mitigated. For CFEPC, the peak displacement gradually increases with the number of cycles at the 3 mm thickness, whereas at the 5 mm thickness it exhibits a non-monotonic trend of first increasing and then decreasing. This may reflect the competition between the interface stability of CFEPC and the progressive deterioration of initial microdefects within the thick layer under cyclic conditioning. In contrast, the peak displacement of PEEPC shows an upward trend at both thicknesses, thanks to the weak interfacial bonding between PEEP and concrete in PEEPC. Unlike fibrous reinforcements whose effectiveness depends on interfacial bond strength, the toughening of PEs relies on particle debonding and subsequent cavitation, which are facilitated rather than hindered by the weak PE-epoxy interface. Consequently, this weak interface is less sensitive to moisture-induced degradation, and the sustained particle debonding mechanism continues to dissipate strain energy throughout cyclic exposure.

To quantitatively assess the flexural toughness of different grouted specimens, the area under the load–displacement curve from the origin to the peak load was integrated and defined as the apparent pre-peak energy absorption (*E_pf_*). Since the displacement was recorded by the machine crosshead rather than by direct midspan measurement, the calculated *E_pf_* is designated as “apparent” to reflect the combined contribution of specimen deflection and machine compliance. This systematic contribution remains consistent across all specimens under identical test conditions, allowing *E_pf_* to serve as a valid comparative index for flexural toughness among different grouted systems. Accordingly, *E_pf_* serves as a comparative index for evaluating the relative flexural toughness among different grouted systems, rather than an absolute material property. Higher *E_pf_* values correspond to enhanced flexural toughness and improved resistance to crack-induced deformation.

**Figure 11:**
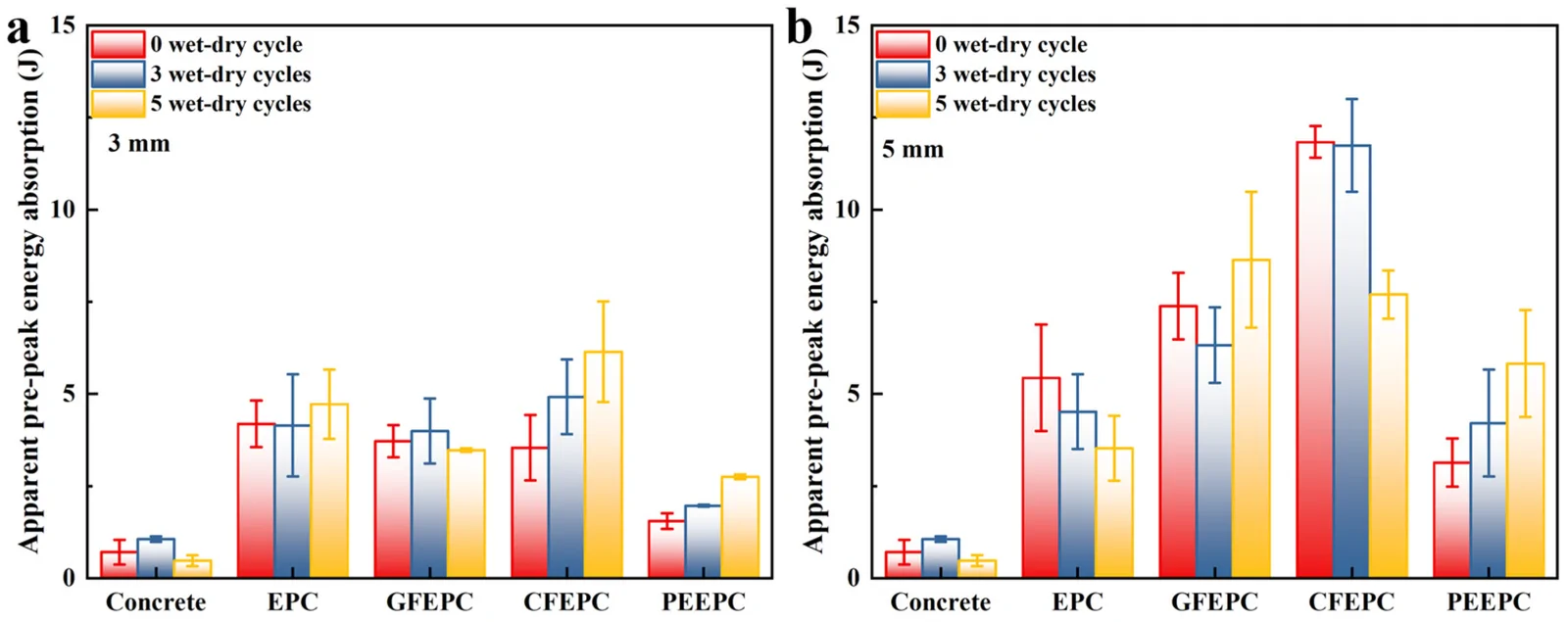
Apparent pre-peak energy absorption of different grouted concrete specimens. (**a**) Grouting layer thickness of 3 mm; (**b**) grouting layer thickness of 5 mm.

As shown in Figure 11, the *E_pf_* values of intact concrete range from 0.47 to 1.06 J, decreasing to 0.47 J after five wet–dry cycles, indicating that pore damage induced by wet–dry cycling exacerbates the brittleness of concrete, which is fully consistent with its brittle fracture characteristics. Notably, a temporary increase in *E_pf_* is observed at three cycles, which is likely attributable to the deposition of late-stage hydration products within the pore structure, temporarily enhancing the toughness of the concrete matrix. The *E_pf_* values of EPC specimens range from 4.14 to 5.43 J, far exceeding those of intact concrete, demonstrating the excellent flexural toughness of the epoxy matrix. At a thickness of 3 mm, from 0 to 3 wet–dry cycles, the *E_pf_* of EPC remains relatively stable; from 3 to 5 cycles, it rises from 4.143 J to 4.721 J, with an increase of 13.9%, suggesting that further crosslinking of the thin resin layer may occur during later-stage cycling, potentially contributing to the observed toughness improvement. However, reinforcement incorporation does not further enhance the toughness of the grouted specimens under the no-cycle condition. The *E_pf_* values of CFEPC and GFEPC are lower than that of EPC, mainly because the limited dispersion space in the thin layer restricts the effectiveness of fiber bridging and stress transfer. In contrast, the *E_pf_* of PEEPC is also lower than EPC, consistent with the relatively low modulus of HDPEs and their limited contribution to flexural load-bearing capacity under static loading. Among these, the *E_pf_* of CFEPC progressively increases to 4.92 J and 6.14 J with wet–dry cycling, indicating that the synergy between the high-modulus carbon fibers and post-curing enables its toughness to surpass EPC. By contrast, although the *E_pf_* of GFEPC and PEEPC increases after cycling, both remain lower than that of EPC. In contrast to the 3 mm case, the *E_pf_* of 5 mm EPC decreases markedly with wet–dry cycling. The concentrated curing exotherm in the thick resin layer generates a higher density of initial microcracks and voids, and repeated swelling-shrinkage stresses promote defect interconnection and propagation, accelerating toughness degradation. At this thickness, fiber toughening becomes apparent. At 0 cycles, the *E_pf_* of GFEPC and CFEPC are 36% and 118% higher than EPC, respectively, and both maintain higher toughness than EPC even after 5 cycles. At the same mass fraction of reinforcement, the 5 mm-thick layer provides larger inter-reinforcement spacing and greater freedom for three-dimensional random distribution, forming a more effective toughening network for fibrous reinforcements. For 5 mm PEEPC, *E_pf_* rises from 3.13 J at zero cycles to 5.82 J at five cycles. The toughening of PE microspheres is governed by a distinct mechanism: stress concentration around the particles induces localized matrix yielding, while particle debonding and subsequent cavitation dissipate substantial fracture energy. For PEEPC, the interface bonding between PEEP and concrete does not rely on strong chemical adhesion [39]. Under wet–dry cycling, the physically dominated weak interface may be less susceptible to moisture-induced chemical degradation, potentially allowing sustained energy dissipation through interfacial micro-slip and contributing to toughness enhancement rather than degradation.

Notably, peak load, peak displacement, and *E_pf_* show non-synchronous trends for some systems, reflecting the different sensitivities of these parameters to microstructural changes. Peak load is more sensitive to interfacial stress transfer and peak displacement to grout matrix stiffness, while *E_pf_* comprehensively reflects the product of both. These distinctions provide richer information for understanding the competition between toughening and degradation in different fiber systems under wet–dry cycling. It should be acknowledged that part of the data in Figure 9, Figure 10 and Figure 11 show significant scatter (relatively large error bars), which is attributable to concrete heterogeneity, manual specimen preparation, and variability in fiber dispersion. With only three replicates per condition, the statistical power is limited; hence, the observed trends are indicative rather than definitive. Nevertheless, the consistent directional trends across multiple conditions, particularly the general superiority of GFEPC in deformation capacity and the thickness-dependent response of CFEPC to wet–dry cycling, provide meaningful comparative insights into the performance of the different reinforcement systems.

### 3.3. Splitting Tensile Behavior Under Multi-Angle Loading

**Figure 12:**
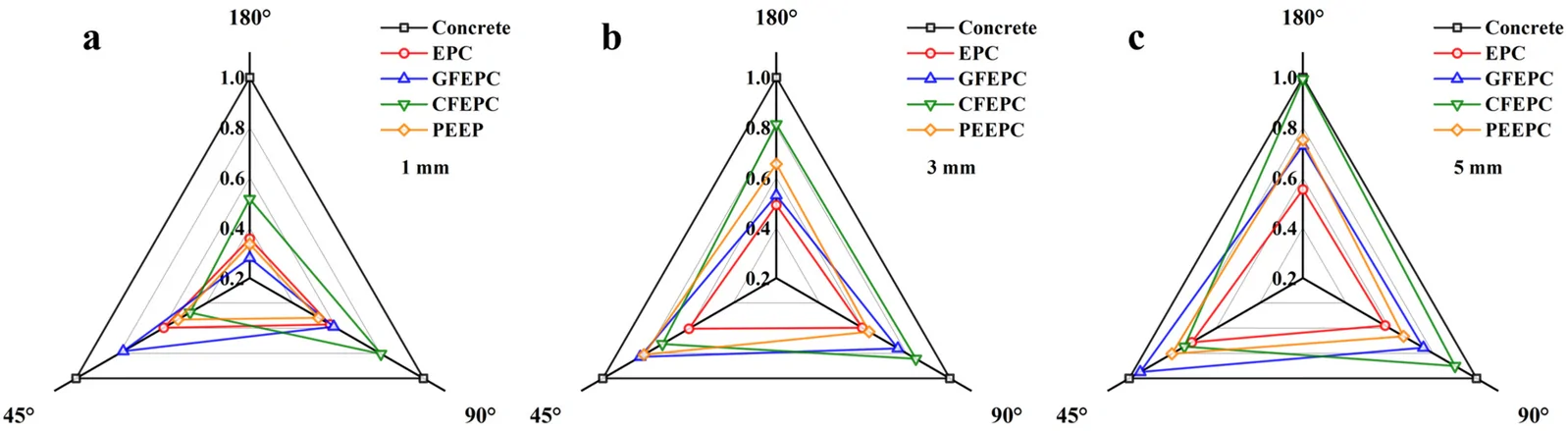
Normalized peak load in splitting tensile tests of grouted concrete under three loading angles. (**a**) Grouting thickness is 1 mm; (**b**) grouting thickness is 3 mm; (**c**) grouting thickness is 5 mm. (The data in the radar charts are normalized by the peak load of intact concrete, with the normalized strength of the intact concrete taken as 1.0).

Brazilian splitting tests were conducted at three loading angles (180°, 90°, and 45°), where 180° denotes loading parallel to the grouting interface. The peak load of all specimens was normalized relative to that of intact concrete, and triangular radar charts were used to compare the load-bearing capacity under different test conditions. For visual clarity, each data point in the radar charts represents the mean value calculated from three replicate specimens, while error bars are not displayed in the figures. The complete dataset, including mean values and standard deviations for all specimens under each condition, is provided in Table A1. As shown in Figure 12, the normalized splitting peak load of the four grouted concretes generally increases with interlayer thickness from 1 to 5 mm under all three loading angles. This indicates that a thicker grout layer optimizes interfacial load transfer, alleviates stress concentration, and thus improves the splitting bearing capacity of grouted specimens. The loading angle affects the tensile bearing capacity of each grouted system. When loading is parallel to the grouting interface (180°), the interface is subjected to a predominantly tensile stress state, which readily causes interfacial debonding. Among all groups, EPC exhibits the lowest normalized peak load under this condition. In contrast, the carbon fibers in CFEPC exert bridging and toughening effects along the loading direction, resulting in excellent splitting tensile performance. At 5 mm thickness, its splitting bearing capacity recovers to 99% of that of intact concrete. For loading perpendicular to the grouting interface (90°), the horizontal tensile stress acts along the interface. Both CFEPC and GFEPC achieve higher peak load than EPC. In contrast, PEEPC exhibits a relatively lower peak load, owing to the weak interfacial bonding between polyethylene microspheres and the epoxy matrix. Under 45° oblique loading, the stress state of the interlayer interface involves a combination of tensile and shear components. Flexible glass fibers effectively relieve stress concentration under oblique bidirectional loading. Accordingly, GFEPC exhibits the highest splitting bearing capacity among the tested systems under this condition, and its peak load reaches 95% of that of intact concrete at 5 mm thickness. Meanwhile, it should be pointed out that the grouting thickness has an undeniable impact on the splitting bearing capacity of the grouted system under a 45° load. At 3 mm thickness (Figure 12b), PEEPC exhibits almost the same peak load as GFEPC, indicating that even weak bonding interfaces can still exhibit high load-bearing capacity at appropriate grouting thicknesses. The advantage of GFEPC becomes more pronounced at 5 mm, indicating that glass fibers require sufficient resin volume to fully develop their crack-deflection capability.

**Figure 13:**
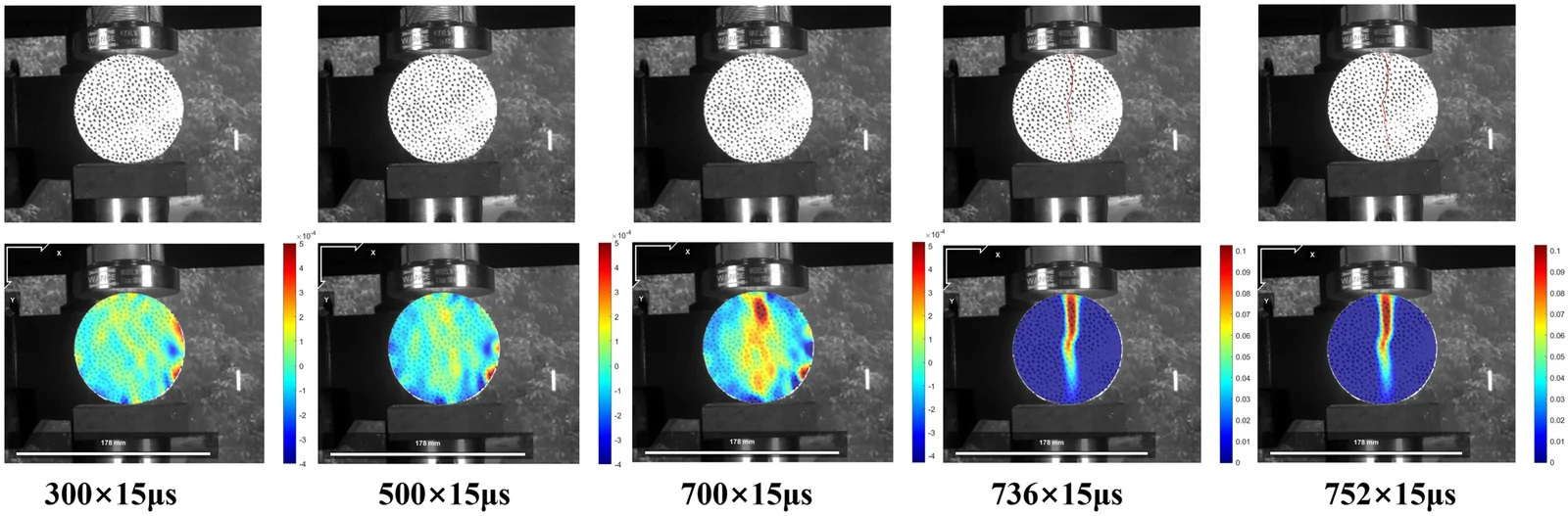
Crack propagation process in the concrete specimen during loading captured by DIC.

Based on the preceding macroscopic strength analysis, specimens with a 3 mm grouting thickness under 45° loading were selected for crack evolution analysis using DIC, including intact concrete, EPC, GFEPC, CFEPC, and PEEPC. Figure 13 presents the specimen surface and corresponding horizontal tensile strain maps of the intact concrete specimen throughout loading. The intact concrete exhibits typical quasi-brittle fracture behavior. At the early loading stage, strain localization and microcracking initiate in the central tension zone. From frame 300 to frame 700, the high-strain zones gradually propagate along the loading direction and eventually coalesce into a continuous main crack at frame 736, leading to the failure of the specimen. The intact concrete undergoes progressive crack initiation and stable propagation before post-peak failure. This behavior originates from microcrack coalescence at aggregate-mortar interfaces. The DIC strain maps clearly reveal this process, showing a transition from scattered strain distribution to localized principal strain bands.

**Figure 14:**
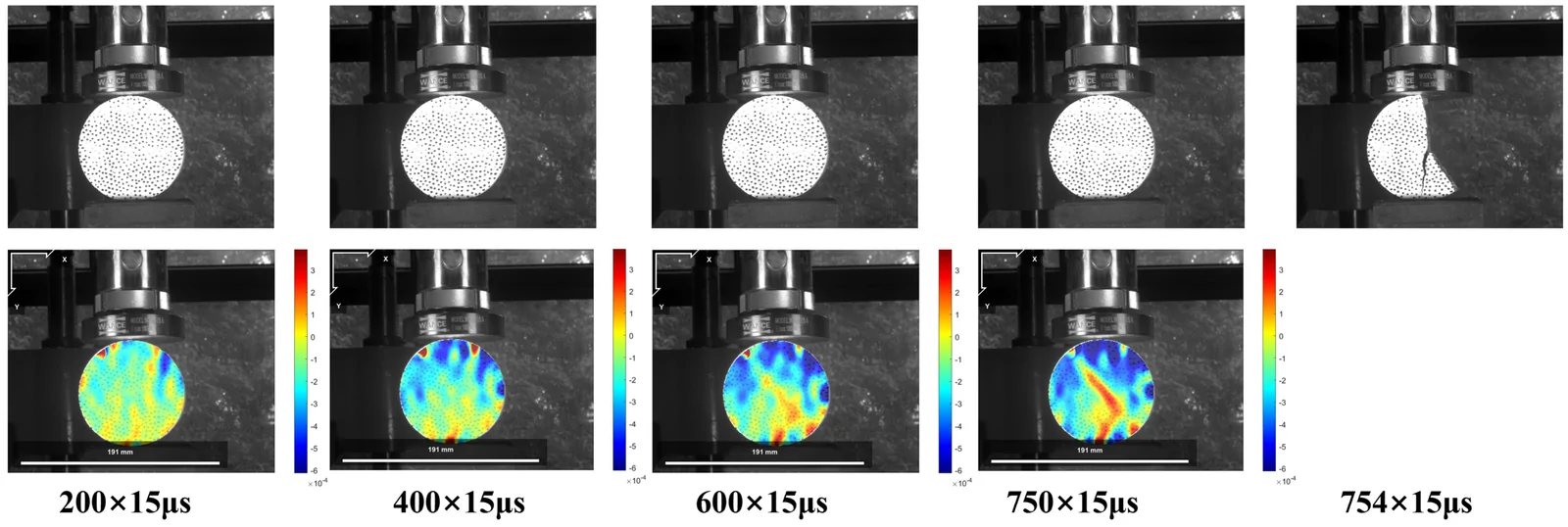
Crack propagation process in the EPC during loading captured by DIC.

In contrast to the progressive quasi-brittle cracking behavior of intact concrete, EPC exhibits distinct linear-elastic brittle fracture characteristics (Figure 14). As revealed by the sequential speckle images and horizontal tensile strain maps, almost no observable stages of slow microcrack initiation or stable propagation are detected throughout the loading process. The specimen experienced an instantaneous penetration splitting failure at frame 754, with a flat fracture surface. The DIC strain maps illustrate the brittle damage evolution of EPC. During the early loading stage (before frame 600), the strain field of the specimen remains uniform, with only sporadic and minor strain concentrations occurring at the edges. No localized high-strain zones develop or propagate prematurely. Unlike the progressive strain localization typically observed in quasi-brittle materials, no precursors of strain localization are detected. When the load approaches the ultimate load (frame 750), a continuous high-tensile strain band spanning the entire cross-section suddenly forms and rapidly coalesces within a very short acquisition interval. At frame 754, a splitting crack appears on the specimen surface, and the specimen undergoes splitting failure. These mesoscopic characteristics indicate that the pure epoxy-grouted system lacks effective energy dissipation mechanisms. Once microcracks initiate, they propagate rapidly and unstably along the weakest path, ultimately triggering instantaneous splitting failure with no observable precursors.

**Figure 15:**
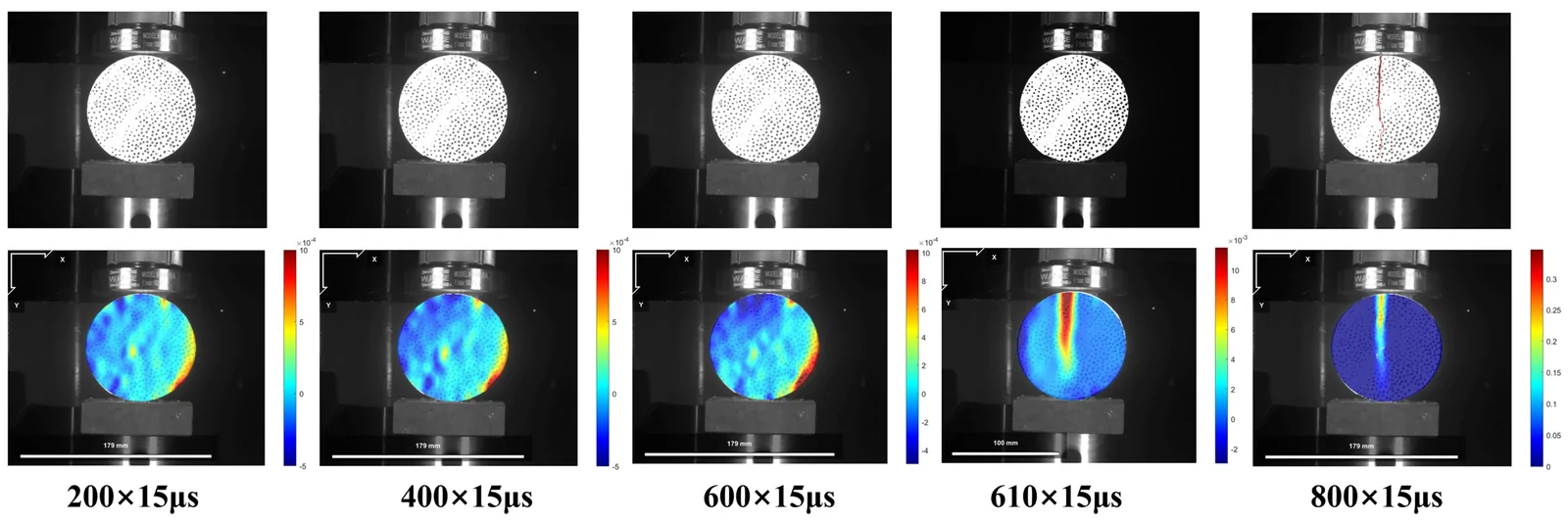
Crack propagation process in the CFEPC during loading captured by DIC.

Figure 15 presents sequential speckle images and horizontal tensile strain maps of CFEPC specimens with a 3 mm grouting thickness under 45° oblique loading. Regarding mesoscopic damage evolution, CFEPC exhibits quasi-brittle fracture behavior similar to that of intact concrete. This behavior is distinctly different from the sudden brittle splitting failure with no observable precursors observed in EPC. During the initial loading stage (first 600 frames), localized strain concentration emerges at the specimen edge. As the applied load increases, the high-strain zones gradually extend along the loading direction, and strain localization further intensifies. At frame 610, a narrow high-strain band forms along the loading axis, while no visible cracks can be seen on the specimen surface. At frame 800, the main strain band coalesces across the entire cross-section, and the macroscopic crack on the specimen surface is fully formed. In contrast to the straight throughgoing main crack of intact concrete, the principal strain band of CFEPC shows slight deflection and rough, undulating boundaries. This phenomenon demonstrates the bridging toughening mechanism of carbon fibers. When the crack tip propagates to the carbon fiber region, the bridging constraint of the fibers deflects the crack growth direction and retards the coalescence of strain localization bands. These overcome the inherent brittle nature of the unmodified epoxy system. The DIC strain maps confirm that the incorporation of carbon fibers prolongs the stable crack propagation stage and imparts progressive quasi-brittle failure behavior to the grouted concrete.

**Figure 16:**
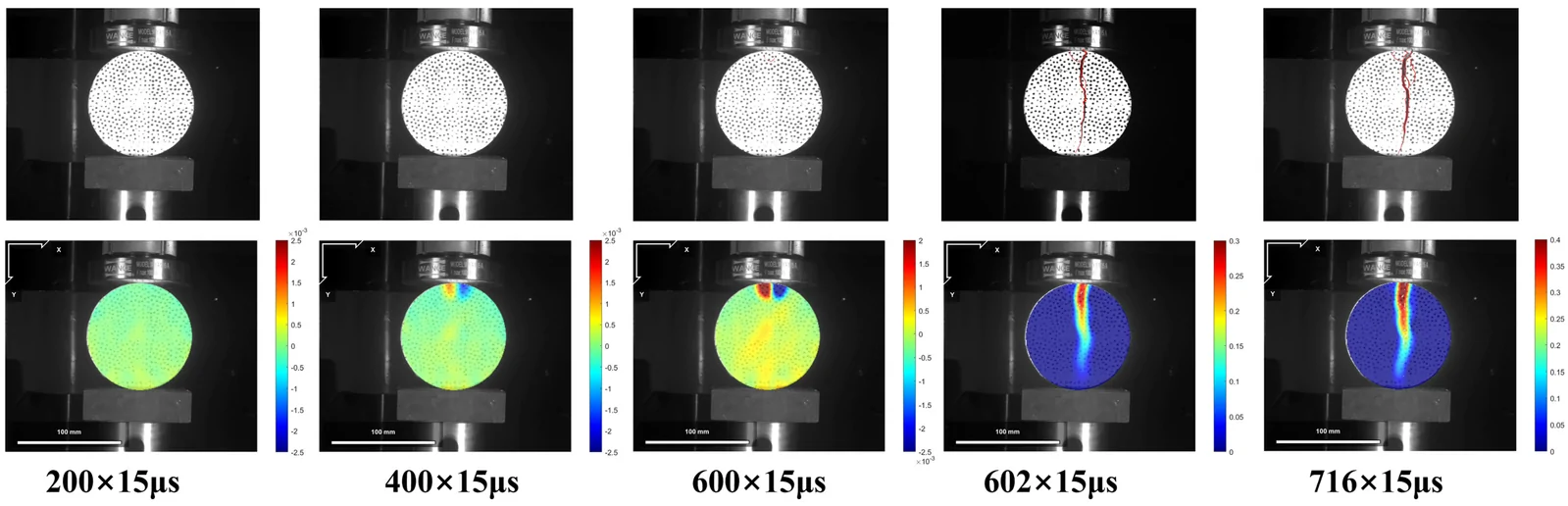
Crack propagation process in the GFEPC during loading captured by DIC.

As shown in Figure 16, GFEPC also exhibits typical quasi-brittle fracture behavior, with ductile toughening effects superior to those of intact concrete and CFEPC. During the early loading stage (before frame 200), the strain field over the entire specimen is uniformly distributed. At frame 400, localized strain concentration first appears at the top loading end. By frame 600, the high-strain zones gradually extend along the loading direction and strain localization progressively intensifies, accompanied by the initiation of microcracks on the specimen surface. From frames 602 to 716, a narrow principal high-strain band gradually forms and coalesces, and a complete macroscopic main crack develops on the specimen surface. In contrast to the straight and smooth main crack in intact concrete and the slightly deflected crack path in CFEPC, GFEPC exhibits a principal high-strain band with rough, undulating boundaries and multiple deflections. Additionally, multiple secondary strain concentration zones emerge around the loading end, corresponding to the simultaneous initiation and branching of several secondary microcracks. This phenomenon indicates that when the crack tip propagates toward the fiber-rich region, collaborative energy dissipation mechanisms, including fiber bridging and fiber-matrix interfacial debonding, effectively retard the rapid propagation of the main crack.

**Figure 17:**
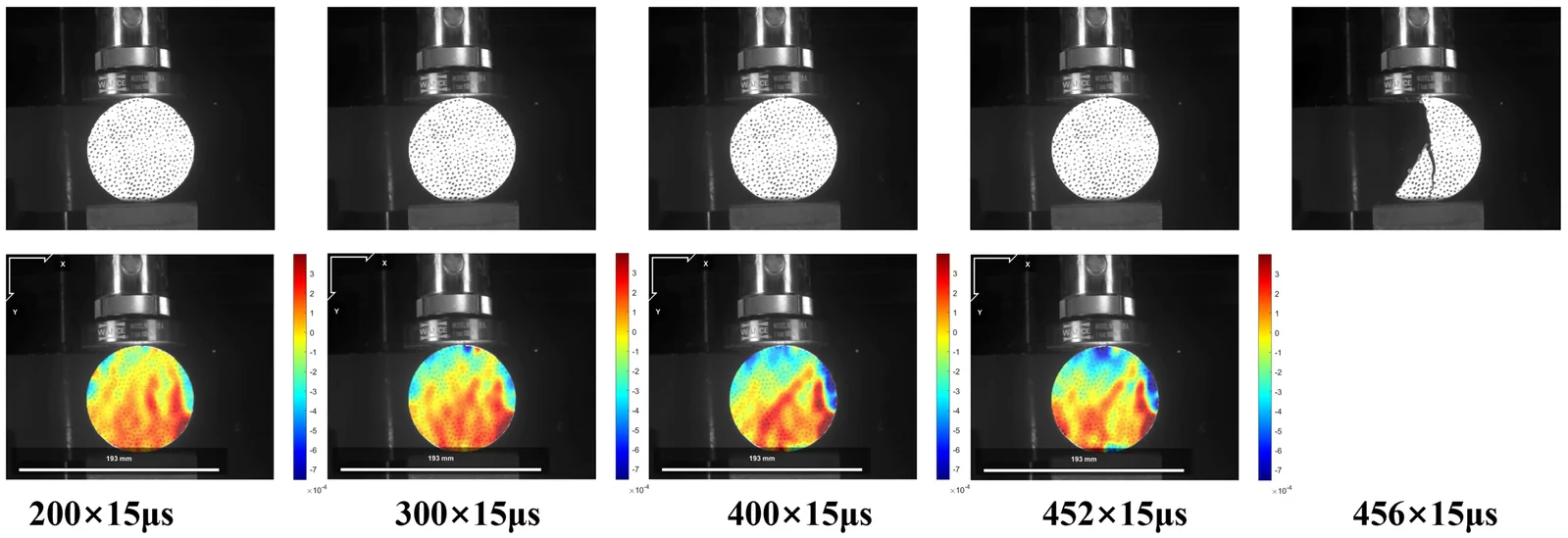
Crack propagation process in the PEEPC during loading captured by DIC.

Unlike EPC, CFEPC, and GFEPC, PEEPC exhibits distinctive and superior ductile quasi-brittle fracture behavior (Figure 17). During early loading (first 300 frames), scattered localized high-strain zones emerge simultaneously at both edges, with diffuse strain distribution and no single dominant strain band. From frames 400 to 452, the various high-strain zones gradually expand outward, continuously adjust their morphology, and intertwine and branch with each other. Until frame 456, splitting failure occurs after sufficient damage accumulation within the specimen. It is evident that PEEPC possesses excellent ductile deformation capacity and crack resistance, which is consistent with the discovery that PEEPC almost reaches the highest bearing capacity consistent with GFEPC under 45° oblique load, as shown in Figure 12b. Unlike rigid carbon fibers and moderate-modulus glass fibers that rely on crack bridging and interfacial stress transfer, the weak interface bonding between PE and epoxy resin promotes the detachment of particles, thereby promoting the diffusion energy dissipation of the entire epoxy matrix. Thus, crack propagation induces extensive particle debonding and void growth rather than direct rupture of the reinforcement, continuously dissipating strain energy. The 45° oblique loading generates both tensile and shear components, and the multi-site diffuse damage with multi-path energy dissipation in PEEPC substantially delays macroscopic instability, resulting in higher splitting tensile capacity under tension-shear interaction.

To further elucidate the interfacial damage evolution mechanisms of grouted specimens under different loading orientations, the DIC strain fields at the peak load instant under the three loading directions are compared. The peak load instant is defined as the moment when the global maximum tensile strain of the specimen reaches its peak value and ceases to increase. All specimens had a grouting thickness of 3 mm.

**Figure 18:**
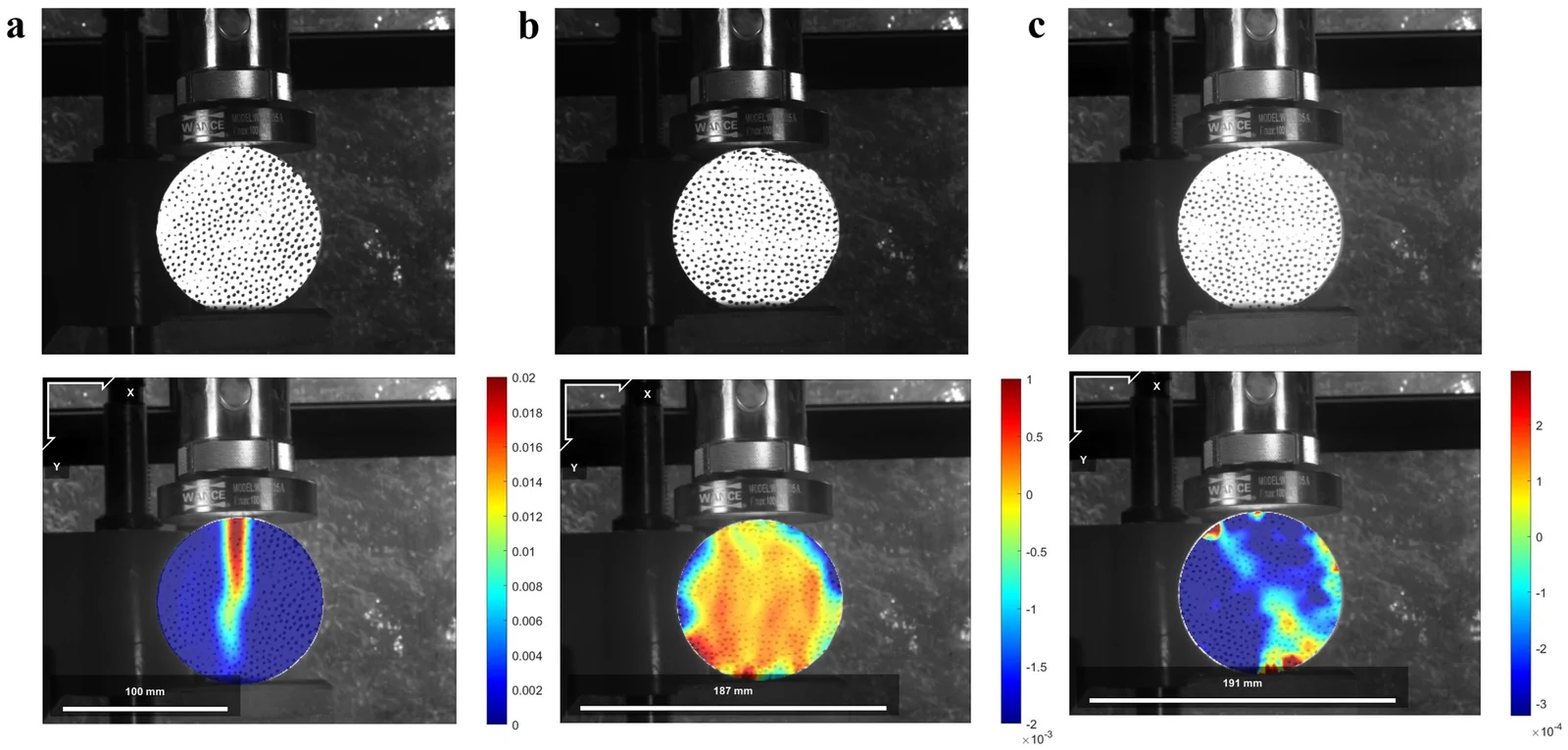
DIC strain contour maps of EPC specimens at the peak load instant under different loading directions: (**a**) 180°; (**b**) 90°; (**c**) 45°.

Figure 18 presents the horizontal tensile strain maps of EPC specimens at the peak load instant under three loading directions, with markedly different strain distributions observed. Under loading parallel to the grouting interface (Figure 18a), a narrow throughgoing high-amplitude tensile strain band forms along the specimen centerline, with strain magnitudes far exceeding those under the other two conditions. The load acts parallel to the epoxy-concrete interface, subjecting it to pure tension. Stress concentrates intensely within the thin interfacial layer, readily inducing a continuous main splitting crack along the centerline, with interfacial tensile failure governing the overall deformation. When loading is perpendicular to the resin layer (Figure 18b), no localized tensile band develops before the peak load. Tensile strain distributes uniformly in a diffuse manner across the specimen. The perpendicular external load generates a bidirectional confinement effect that impedes localized crack initiation and propagation. Under 45° oblique loading (Figure 18c), tensile strain appears as scattered isolated patches without obvious stress concentration, exhibiting the lowest peak tensile strain magnitude. However, as shown in Figure 14, although no throughgoing principal strain band forms at the peak instant, EPC still undergoes complete splitting failure immediately after the peak load. This is ascribed to the inherent linear-elastic brittle nature of the unmodified epoxy matrix. While the oblique loading alleviates local stress concentration via its tensile and shear components, reducing the global strain level and avoiding premature strain localization, it cannot dissipate the fracture energy released during crack propagation. Once the peak load is reached, multiple microcracks initiate at pre-existing interfacial defects and propagate rapidly and unstably along weak paths along the epoxy-concrete interface, soon coalescing into a main crack and resulting in instantaneous splitting failure without progressive crack growth or appreciable precursors.

**Figure 19:**
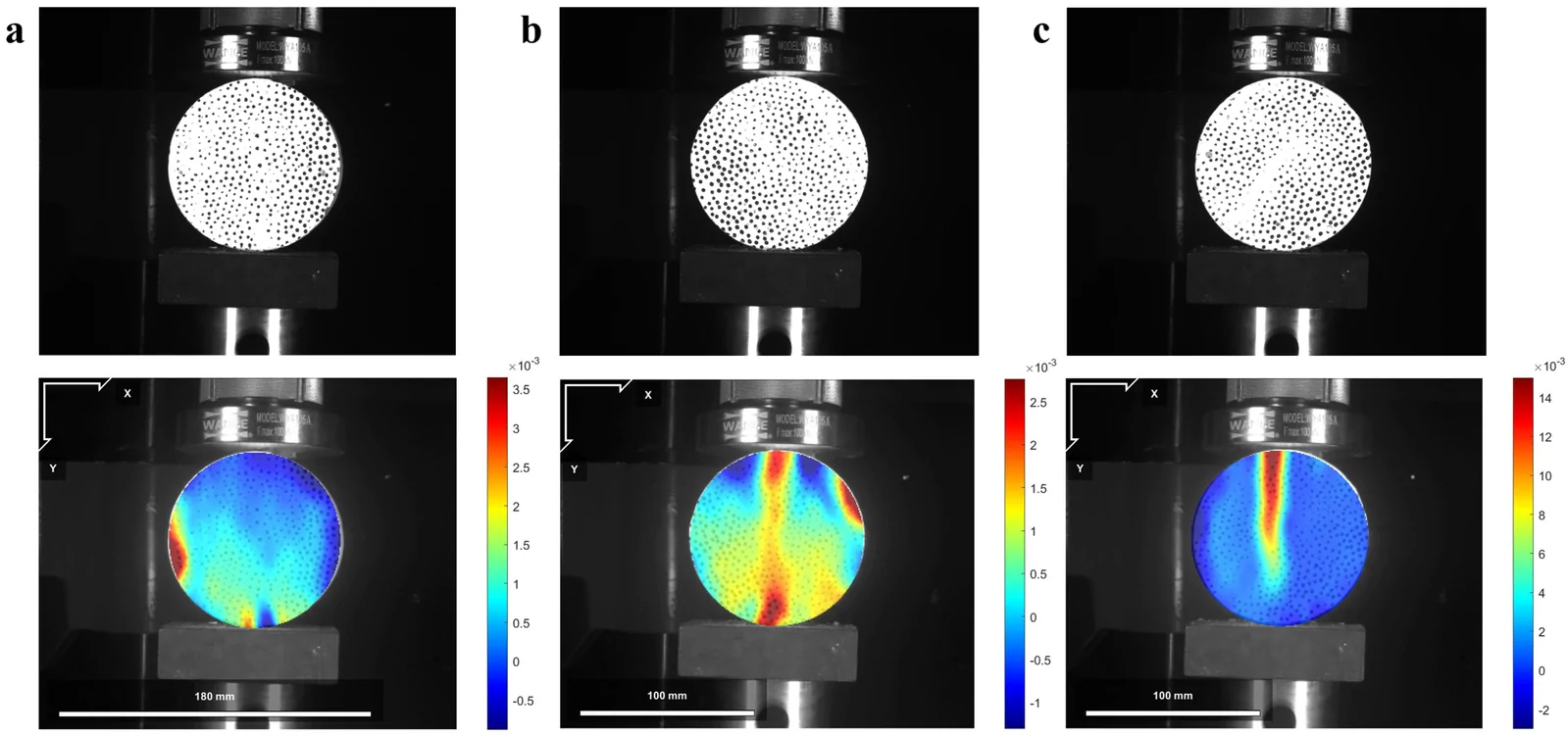
DIC strain contour maps of CFEPC specimens at the peak load instant under different loading directions: (**a**) 180°; (**b**) 90°; (**c**) 45°.

The DIC strain contour maps of CFEPC at the peak load instant under the three loading directions differ markedly from those of EPC. Under 180° loading (Figure 19a), high tensile strain is scattered only in a local area at the lower-left corner edge of the specimen, and no throughgoing main tensile strain band develops along the loading diameter. The peak tensile strain is only 3.5 × 10^−3^. Under pure tension, the bridging effect of carbon fibers effectively disperses interfacial stress concentration, suppresses the formation of a single continuous main crack, and reduces the overall strain level. Under 90° loading (Figure 19b), a medium-amplitude throughgoing tensile band forms at the midline of the specimen, with a peak tensile strain of 2.5 × 10^−3^ across the field. No extreme localized high-strain hotspots are observed. Under the combination of tensile and shear components (Figure 19c), the specimen exhibits an extremely narrow high-gradient throughgoing tensile strain band along the loading centerline, with a peak tensile strain of 14 × 10^−3^, significantly exceeding that under the other two loading conditions. The oblique load couples tensile and shear forces, leading to pronounced stress concentration at the crack tip in CFEPC. Although carbon fibers deflect the crack path and provide toughening and energy dissipation, the superposition of tensile and shear stresses amplifies local strain concentration in the midline region. Both the carbon fibers and the epoxy matrix are inherently brittle, resulting in limited plastic deformation capacity. Stress becomes highly concentrated and is released abruptly at the weakest section, forming an extremely narrow strain localization band. Consequently, the peak strain of CFEPC under 45° loading is much higher than that of EPC.

**Figure 20:**
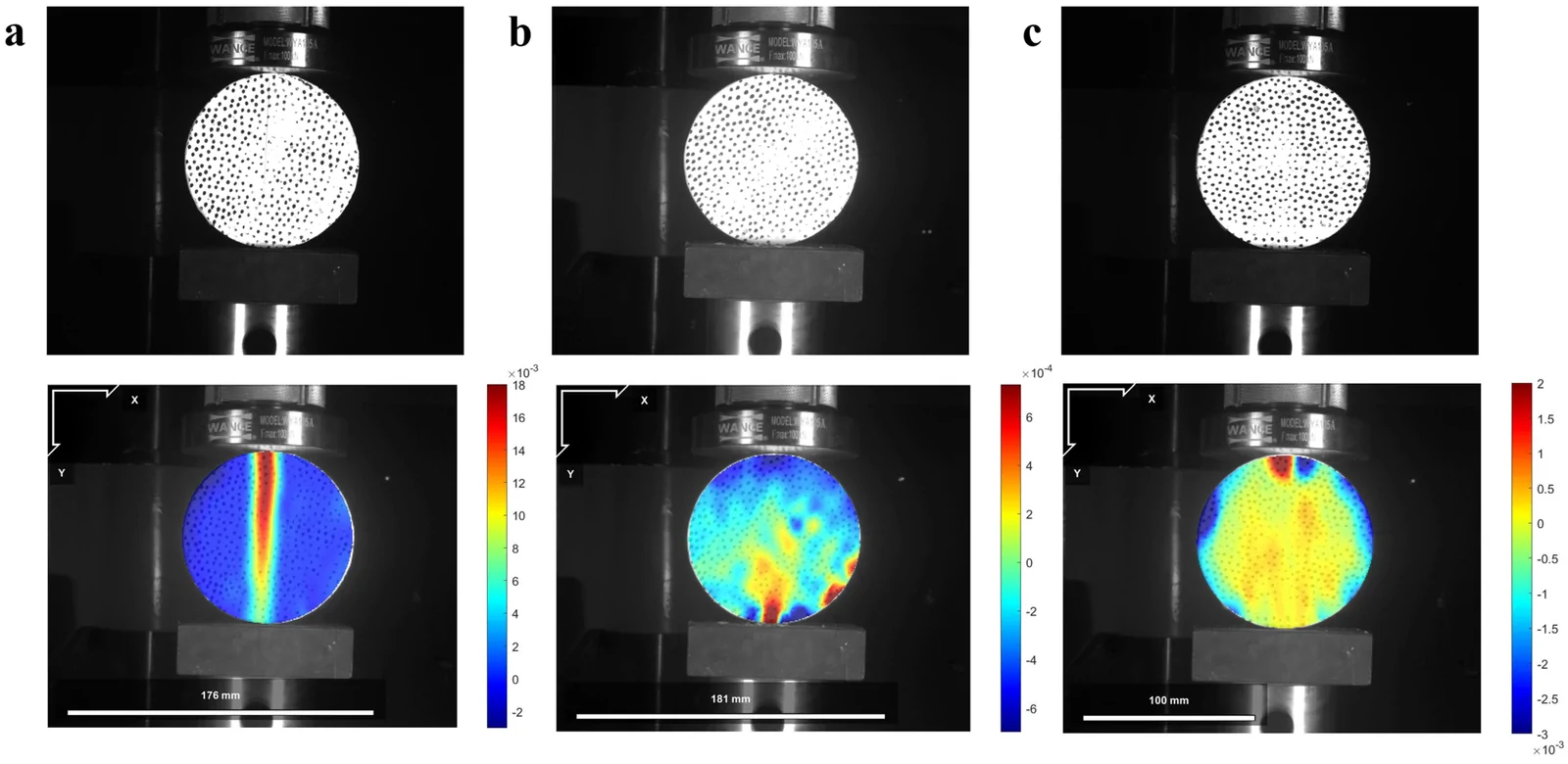
DIC strain contour maps of GFEPC specimens at the peak load instant under different loading directions: (**a**) 180°; (**b**) 90°; (**c**) 45°.

Figure 20 presents the horizontal tensile strain maps of GFEPC at the peak load instant. The strain distribution of GFEPC under the three loading directions resembles that of EPC. When loaded parallel to the grouting interface (Figure 20a), a throughgoing high-amplitude tensile strain band forms in the specimen, with tensile stress concentrated along the interface, ultimately inducing splitting failure. When loaded perpendicular to the grouting interface (Figure 20b), the strain exhibits a scattered low-amplitude distribution in the bottom region, with a strain amplitude lower than that of EPC and CFEPC. Under 45° loading, GFEPC exhibits a more uniform tensile strain distribution throughout the specimen than EPC, effectively suppressing severe stress concentration (Figure 20c). This indicates that the glass fiber-reinforced system possesses superior crack resistance under oblique loading.

**Figure 21:**
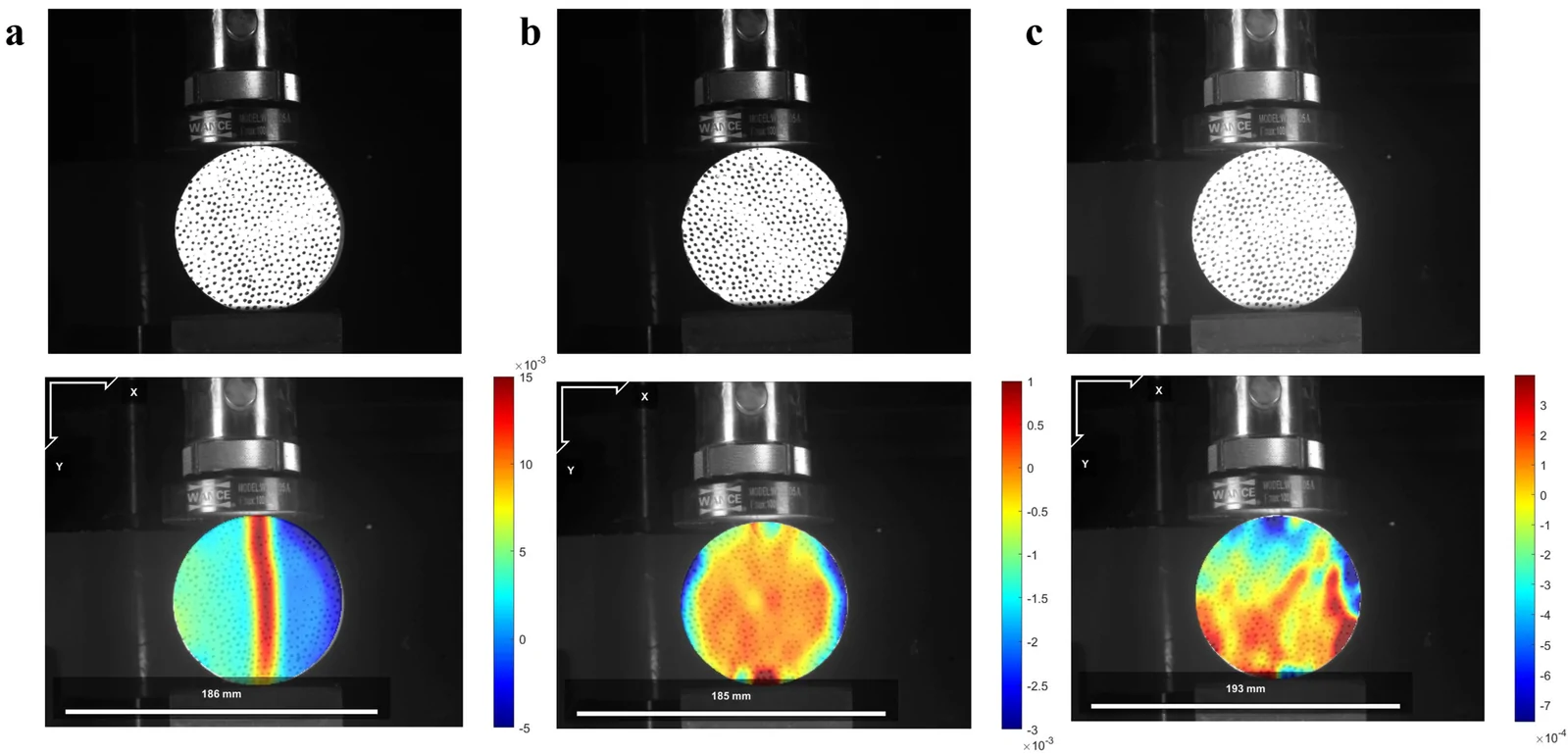
DIC strain contour maps of PEEPC specimens at the peak load instant under different loading directions: (**a**) 180°; (**b**) 90°; (**c**) 45°.

The horizontal tensile strain maps of PEEPC specimens at the peak load instant are presented in Figure 21. Similar to those of EPC and GFEPC, when loaded parallel to the grouting interface (Figure 21a), a throughgoing tensile strain band forms in the specimen. However, the strain band is wider with a lower peak strain amplitude. Under both perpendicular and oblique loading, no throughgoing main crack forms. In contrast, under perpendicular loading (Figure 21b), the specimen exhibits widespread uniform tensile strain zones, accompanied by more extensive tensile damage and higher overall deformation. Under 45° oblique loading (Figure 21c), a visible local high-strain zone appears near the bottom of the specimen, and the stress concentration phenomenon is more significant than that in EPC and GFEPC.

### 3.4. Interfacial Failure Mechanisms Revealed by DIC-Based Strain Field Analysis

The DIC strain field analysis across the three loading directions and four grouted systems reveals three characteristic failure patterns, governed by the interplay between interfacial bond strength and stress state. Prior work has established that the CFEP system exhibits the strongest interfacial bonding with concrete, characterized by synchronous fracture of fibers and matrix upon interface separation. The GFEP system shows moderate bonding strength, with interfacial failure involving fiber pull-out and limited slip. The PEEP system exhibits the weakest bonding, where the weak PE-epoxy interface facilitates particle debonding under loading [39]. These interfacial characteristics provide the mechanistic foundation for interpreting the DIC-observed strain localization behaviors. Under 180° loading (parallel to the interface), the interface is subjected to a predominantly tensile stress state, with failure governed by interfacial bond strength. EPC, lacking reinforcement, shows the weakest interface with rapid debonding. CFEPC achieves the highest bond strength and the most uniform strain distribution, as the strong interface transfers stress effectively and suppresses strain localization. PEEPC, despite its weak bonding, enables extensive interfacial micro-slip that broadens the strain band and delays coalescence under tension. Under 90° loading (perpendicular to the interface), horizontal tensile stress acts parallel to the grouted layer, with load-bearing capacity governed by the tensile strength and modulus of the grouting material. The ultrahigh modulus of CFEPC enables efficient stress transfer from the matrix, producing a broad strain band that indicates uniform damage distribution and effective suppression of localized stress concentration. GFEPC and PEEPC, by contrast, exhibit no throughgoing strain bands and show only local low-amplitude strain concentrations due to their lower moduli. Under 45° oblique loading, a combination of tensile and shear stress components acts on the interface, where deformation compatibility becomes the governing factor. The strong and rigid CFEPC interface, effective under pure tension, shows limited deformability under coupled loading, leading to abrupt stress concentration and release at the weakest section, with an extremely narrow strain localization band. GFEPC, with moderate bonding that allows limited slip, offers balanced strength and compatibility, excelling under tension-shear interaction. PEEPC, with the weakest bonding, enables sustained particle debonding, dissipating energy through diffuse multi-site damage and substantially delaying macroscopic instability.

In summary, the DIC strain field analysis, combined with the interfacial bonding characteristics of each system, confirms a direct correlation between interfacial micromechanical properties and macroscopic failure paths. Strong interfaces excel in stress transfer and resistance under tensile-dominated loading but are prone to sudden failure under tension-shear interaction. Moderate interfaces offer balanced strength and compatibility, performing best under tension-shear interaction. The unique particle debonding mechanism of PE and the interfacial slip mechanism of GF can both increase energy dissipation and delay failure. These findings underscore that reinforcement selection should be guided by the anticipated dominant loading mode in service.

## 4. Conclusions

This study systematically investigates the mechanical performance and short-term wet–dry performance of concrete grouted with carbon fiber-reinforced, glass fiber-reinforced, and polyethylene microsphere-reinforced epoxy. The effects of reinforcement type, grouting thickness (1, 3, 5 mm), multi-angle splitting tensile loading (180°, 90°, 45°), and successive wet–dry cycles (0, 1, 3, 5 cycles) are evaluated via compression, three-point bending, and DIC strain field monitoring. Based on the experimental findings, the following conclusions can be drawn:

(1) The incorporation of reinforcements mitigates the brittleness of traditional pure epoxy grout layers. The CFEPC exhibited the most outstanding compressive load-bearing capacity among the fiber-reinforced systems. Furthermore, a distinct size effect regarding the grouting thickness was identified. Among the tested thicknesses, the 3 mm grouting thickness proved to be the optimal dimension, effectively balancing uniform reinforcement dispersion and resin wetting while avoiding the elevated internal shrinkage stresses and defect accumulation observed in thicker grouting layers.
(2) While pure epoxy grout layers are highly susceptible to brittle catastrophic failure, reinforcement addition successfully transforms the structural response to ductile. PE and GF systems demonstrated exceptional advantages in flexural deformation capacity. Notably, the GFEPC increased the ultimate displacement of intact concrete and EPC by at least 150% and 30%, respectively, effectively delaying the failure of the tension zone.
(3) The splitting failure mechanism of the interlayer grouted concrete specimens is directly governed by the interfacial bond strength of each system. CFEPC with a strong-bonding interface exhibits good crack resistance under tensile-dominated loading but tends to fail in a more brittle manner under tension-shear interaction. GFEPC with a moderate-bonding interface shows favorable performance under tension-shear interaction. PEEPC with a weak-bonding interface delays failure through sustained particle debonding and energy dissipation.
(4) Wet–dry cycles trigger moisture-induced plasticization and thermal mismatch stresses, which severely degrade the unreinforced epoxy-concrete interface. However, reinforced epoxy grout layers maintained high interfacial integrity. After five wet–dry cycles, the pure epoxy specimens suffered a drastic strength reduction, whereas both GFEPC and CFEPC maintained high bearing capacity and exhibited excellent interfacial stability, which can resist environmental changes.

It is acknowledged that the specimens use idealized uniform-thickness grouting layers between two concrete parts, unlike actual cracked and grout-injected concrete, and that no cracked and un-grouted controls were included, as the primary objective was comparative evaluation of reinforcement toughening rather than quantification of absolute capacity recovery. Nevertheless, the systematic comparison across three reinforcement types, three grouting thicknesses, three loading angles, and multiple wet–dry cycles provides robust mechanistic insights into reinforcement-dependent toughening pathways and interfacial failure modes, which may provide preliminary guidance for the design of reinforced epoxy grout systems, pending validation under broader conditions. Future studies with cracked controls and more realistic crack geometries are warranted to further validate these findings. In addition, the wet–dry cycling regime in this study was limited to five cycles, which captures early-stage degradation trends under short-term conditioning but does not represent long-term service exposure. Extended cycling and real-environment validation are necessary to fully assess the long-term performance of these reinforced epoxy grout systems.

## Figures and Tables

**Table 1:** Mix proportion of concrete.

Cement (kg/m^3^)	River Sand (kg/m^3^)	Crushed Stone (kg/m^3^)	Fly Ash (kg/m^3^)	Polycarboxylate Superplasticizer (kg/m^3^)	Water (kg/m^3^)	Water-to-Binder Ratio (W/B)
400	719	1126	50	2.7	156	0.35

**Table 2:** Basic physical and mechanical properties of reinforcing materials.

Reinforcing Materials	Morphology	Length (μm)	Diameter (μm)	Aspect Ratio	Density (g/cm^3^)	Tensile Strength (MPa)	Elasticity Modulus (GPa)
GF	fiber	350	11–17	21–32	2.54	1400–2100	70
CF	fiber	50	7	7.1	1.75	≥4300	250–280
PE	microsphere	-	60–80	-	0.92	11	0.8–1.2

## Data Availability

The original contributions presented in this study are included in the article. Further inquiries can be directed to the corresponding author.

## Appendix A

**Table A1:** The average and standard deviation of the peak load of all specimens under various conditions in splitting tensile tests.

Sample	Loading Angles (°)	Resin Layer Thickness (mm)	Peak Load (kN)	
			Average	Standard Deviation
Intact concrete	—	—	37.926	3.074500285
EPC	180	1	13.540	1.5981
		3	18.642	4.3925
		5	20.993	4.6436
	45	1	22.614	2.2161
		3	22.880	1.01823
		5	27.000	5.3316
	90	1	21.591	2.7867
		3	22.605	1.4602
		5	21.951	6.8865
GFEPC	180	1	10.631	2.5661
		3	20.095	2.2029
		5	27.607	2.1475
	45	1	29.688	4.9908
		3	31.394	2.2104
		5	35.999	2.3695
	90	1	22.263	6.5811
		3	28.831	2.7641
		5	28.636	7.6254
CFEPC	180	1	19.552	2.1828
		3	30.840	2.0365
		5	37.675	0.8294
	45	1	18.000	5.9312
		3	27.505	1.2056
		5	28.301	0.6725
	90	1	30.498	5.5267
		3	31.993	3.0427
		5	34.160	6.4891
PEEPC	180	1	12.710	0.6435
		3	24.829	2.8122
		5	28.484	7.8213
	45	1	20.117	3.6225
		3	30.724	4.4258
		5	30.481	5.7813
	90	1	19.5908	2.1236
		3	23.822	6.9438
		5	25.175	2.9281
